# Bedtime vs Morning Antihypertensive Medications in Frail Older Adults: The BedMed-Frail Randomized Clinical Trial

**DOI:** 10.1001/jamanetworkopen.2025.13812

**Published:** 2025-05-12

**Authors:** Scott R. Garrison, Erik R. E. Youngson, Danielle A. Perry, Farah N. Campbell, Christina S. Korownyk, Lee A. Green, Michael R. Kolber, Jessica E. M. Kirkwood, G. Michael Allan, Roni Y. Kraut, Finlay A. McAlister, Michael D. Hill, Jeffrey A. Bakal

**Affiliations:** 1 Pragmatic Trials Collaborative, University of Alberta, Edmonton, Alberta, Canada; 2 Department of Family Medicine, University of Alberta, Edmonton, Alberta, Canada; 3 Provincial Research Data Services, Alberta Health Services, Edmonton, Alberta, Canada; 4 Alberta Strategy for Patient Oriented Research Support Unit (AbSPORU), Edmonton, Alberta, Canada; 5 College of Family Physicians of Canada, Mississauga, Ontario, Canada; 6 Division of General Internal Medicine, Department of Medicine, University of Alberta, Edmonton, Alberta, Canada; 7 Department of Clinical Neurosciences, Cumming School of Medicine, University of Calgary, Calgary, Alberta, Canada

## Abstract

**Importance:**

The effect of antihypertensive administration time on major adverse cardiovascular events is unclear, and has never been studied in frail older adults, for whom risks and benefits may differ from the general population.

**Objective:**

To determine, in frail seniors, the effect of bedtime vs morning administration of antihypertensive medications on major cardiovascular events and death, as well as on potentially ischemic or hypotensive adverse events.

**Design, Setting, and Participants:**

This multicenter, open-label, pragmatic randomized clinical trial recruited from 13 continuing care facilities in Canada (17 wards; 14 long-term care and 3 supportive-living wards) from May 25, 2020, to September 18, 2023, with follow-up until February 29, 2024. Residents with hypertension and at least 1 once-daily antihypertensive medication were included. Data analysis was from March to August 2024.

**Interventions:**

Patients were randomized 1:1 to taking all once-daily antihypertensive medications either at bedtime (intervention) or per usual care control (largely morning use).

**Main Outcomes and Measures:**

The composite primary outcome was first occurrence of all-cause death or either hospitalization or emergency department (ED) visit for stroke, acute coronary syndrome, or heart failure. All-cause unplanned hospitalization or ED visits, falls and fractures, decubitus ulcers, and worsening cognition or behavioral problems were also assessed.

**Results:**

A total of 776 older adults (median [IQR] age, 88 [81-92] years; 562 female [72.4%]; 664 [85.6%] with some degree of dementia; 367 [47.3%] with diabetes; 307 [39.6%] with coronary artery disease) were randomized to bedtime (394 participants) vs usual care (382 participants) administration and were followed-up for a median (IQR) of 415 (251-735) days. Of 320 primary outcome events, 293 (91.6%) were deaths; there was no difference in primary outcomes for bedtime vs usual care in a modified intention-to-treat analysis (29.4 vs 31.5 events per 100 patient-years; adjusted hazard ratio [aHR], 0.88; 95% CI, 0.71-1.11; *P* = .28). Other outcomes were similarly no different between groups, excepting all-cause unplanned hospitalization and ED visits, which favored bedtime (22.6 vs 30.0 events per 100 patient-years; aHR, 0.74; 95% CI, 0.57-0.96; *P* = .02).

**Conclusions and Relevance:**

In this randomized clinical trial of antihypertensive medication timing, switching antihypertensives to bedtime failed to reduce a composite of death or major cardiovascular events that were primarily all-cause death, and had no effect on potentially ischemic and hypotensive adverse events, suggesting that in a population of frail older adults, administration time had little or no influence on the benefits and risks of antihypertensive medication.

**Trial Registration:**

ClinicalTrials.gov Identifier: NCT04054648

## Key Points

**Question:**

In frail older adults, does bedtime administration of blood pressure lowering medication, compared with conventional morning use, reduce a composite of death or major cardiovascular events?

**Findings:**

In this randomized clinical trial of 776 nursing home residents with hypertension in Canada, bedtime administration of antihypertensive medications had no effect on death or major cardiovascular events (29.4 per 100 patient-years vs 31.5 per 100 patient-years in usual care, largely morning antihypertensive users), nor was there a difference in falls or fractures, decubitus ulcers, or worsening cognition or behavioral problems.

**Meaning:**

In frail older adults, administration time had no effect on benefits and risks of blood pressure lowering medication, suggesting individual patient preferences and circumstances should govern the timing of blood pressure medication.

## Teaser

This randomized clinical trial compares bedtime vs morning administration of antihypertensive medications and its effect on major cardiovascular events and death among older frail adults.

## Introduction

Blood pressure (BP) has a circadian rhythm with lower pressures overnight,^1,2^ and sleep-time BP, compared with daytime BP, better predicts cardiovascular risk, which suggests elevated BP might convey greater cardiovascular risk when occurring at night.^3,4,5^ Given antihypertensive medications might preferentially lower overnight BP when administered at bedtime,^6^ switching antihypertensives to bedtime could hypothetically lower cardiovascular risk. Although 2 randomized clinical trials (RCTs) reported large reductions in major adverse cardiovascular events with bedtime administration compared with conventional morning use (a 61% reduction in Hermida et al^7^ and a 45% reduction in Hermida et al^8^), a subsequent RCT (Mackenzie et al^9^) and a large observational study^10^ reported no difference in mortality and morbidity regardless of administration time. Of these 3 RCTs,^7,8,9^ only Mackenzie et al^9^ reported adverse effects. Collectively, this leaves uncertainty surrounding benefits and harms of bedtime antihypertensive use.

Eligibility criteria and recruitment methods of therapeutic RCTs tend to exclude those who are elderly, highly comorbid, physically frail, or cognitively impaired.^11,12,13,14^ This is problematic given that risks and benefits could differ in frail seniors,^15^ and that bedtime antihypertensives might further lower the lowest BP of the day, potentially promoting ischemic or hypotensive complications such as decubitus ulcers,^16,17^ hypotension-related falls and fractures,^18,19^ or worsening cognition in patients with dementia.^20,21^ They might also worsen night-time agitation and confusion in patients with dementia if the normal lowering of evening BP plays any role in modulating that common presentation (sundowning syndrome).^22,23,24^ Given that benefit demonstrated in younger and healthier populations is often generalized to older and more complex populations poorly represented in the original research, trials assessing both benefit and harm in frail and highly comorbid populations, such as continuing care (nursing home) residents, are critical. In the BedMed-Frail antihypertensive timing trial, we randomly allocated continuing care residents in Canada to take all once-daily antihypertensive medications either at bedtime, or per usual care (largely morning use), and examined differences in mortality and cardiovascular morbidity, and potential hypotensive and ischemic adverse effects.

## Methods

### Trial Design and Organization

BedMed-Frail was an open-label, 1:1 parallel RCT with outcomes drawn from routinely collected administrative health data. The protocol and analysis plan have been published (Supplement 1).^25^ Recruitment ran from May 25, 2020, to September 18, 2023, and observation continued until February 29, 2024. The trial was multicenter, involving 17 continuing care wards within 13 facilities in Alberta, Canada (eAppendix 1 in Supplement 2). The University of Alberta health ethics review board provided ethical approval, and the University of Alberta Pragmatic Trials Collaborative^26^ oversaw the trial, which was registered at ClinicalTrials.gov (NCT04054648). The study followed the Consolidated Standards of Reporting Trials (CONSORT) reporting guideline.^27^

### Participants

Residents at participating continuing care wards were included if they had 2 or more health system encounters with a hypertension diagnosis from 2002 onwards and used 1 or more once-daily antihypertensive medications. Loop-diuretics were not considered antihypertensives. We excluded those with prior glaucoma diagnosis or glaucoma medication, given that nocturnal hypotension is associated with optic neuropathy and visual deterioration in patients with glaucoma.^28,29,30^ We also excluded those hospitalized at the time of randomization and those opting out of the trial. We did not exclude those with other potential indications for antihypertensive use (eg, heart failure or angina) to maximize generalizability and because antihypertensive timing might still modulate risk or benefit in such individuals. Most residents were on long-term care wards (88.3%), which provide the highest level of care. Residents with less care requirements were on designated supportive living wards (DSL4; 7.9%), or DSL4 wards with enhanced dementia care (DSL4-D; 3.9%).^25^

A minimum of 30 days prerandomization, all residents, families, and physicians received written information describing the trial via facility e-mail or letter-mail, or within new resident information packages; this included contact information for opting residents out of the trial for any reason. Opt-out consenting (occurring prerandomization) was considered ethical because (1) RCT evidence at the time (Hermida et al^7^) suggested bedtime antihypertensive use provided better health outcomes, (2) unless physicians specified otherwise, antihypertensives were administered by each facility in the morning by default, and (3) antihypertensive timing in the control group would not change (ie, any evening use in controls would continue).

### Setting and Databases

Alberta’s publicly funded health care system maintains linkable databases tracking all usual care health care interactions for all 4.6 million residents; this includes the most responsible diagnosis submitted by acute care physicians along with emergency department (ED) billings and hospital discharge dictations, community physician diagnoses, prescriptions dispensed, and vital statistics—with validated coding algorithms for outcomes of interest.^31,32,33^ It also includes usual care clinical nursing data for continuing care residents, recorded in the Resident Assessment Instrument–Minimum Dataset 2.0 (RAI-MDS).^34^ RAI-MDS tracks key health impairments to assist nurses and allied health professionals with care planning, and includes (among others) data on falls, continence, skin ulceration, cognition, depression and anxiety, and problem behaviors. RAI-MDS data are collected on admission by nurses providing direct care, and updated by them, typically every 3 months, or upon major changes in health status. Alberta Health Services, 1 of 2 stewards of these databases, facilitated the trial. Relevant, but unavailable, were BP measurements. However systematic review and crossover trial evidence suggest daytime BP is not altered by switching antihypertensive medication to bedtime.^35,36^ In each facility, pharmacists bubble-packed medications by administration time and forwarded resident-specific medication cards to the ward for administration by nurses.

### Interventions

Once through the 30-day opt-out period, an Alberta Health Services analyst (E.R.E.Y.) with no patient contact (1) used administrative health data to identify eligible residents, (2) accessed an investigator-maintained REDCap^37^ database to identify and exclude opted-out residents, (3) centrally randomized and allocated remaining eligible residents to intervention or control (no stratification or blocking), and (4) provided an allocation list to facility pharmacists who bubble-packed medications. Once randomizations began at a facility, new residents were similarly randomized on a monthly basis.

#### Intervention

The intervention group had administration of all once-daily antihypertensives while being readied for bed. Antihypertensives taken more than once daily were unchanged and only 1 timing change per week was recommended. Subsequent antihypertensive prescribing was per usual care.

#### Control

The control group had no change to antihypertensive timing, effectively morning administration for the vast majority. Physicians and pharmacists could adjust medication timing whenever clinically indicated (no special monitoring and both groups treated equally). If bedtime administration proved problematic, pharmacists were asked to consider switching that medication to dinnertime, rather than returning to morning use. Although initially concerned diuretic users might experience adherence-threatening nocturia, we included them given that (1) 3 previous RCTs^7,8,9^ all enrolled diuretic users; (2) extra urine production from thiazide-like and potassium-sparing diuretics would likely be modest; (3) many long-term care residents are incontinent and rely on incontinence briefs with or without scheduled overnight changes (making extra urine volume less noticeable or problematic); and (4) excluding common medications would limit recruitment and generalizability.

### Outcomes

The primary outcome was time to a composite of all-cause death or either hospitalization or ED visit for acute coronary syndrome, stroke, or heart failure. Secondary outcomes included each primary outcome component individually, all-cause unplanned hospitalization or ED visit, nonvertebral fracture and (per the RAI-MDS report closest to 135 days after randomization) fall in the last 30 days, urinary incontinence (≥2 times per week), skin ulceration (stage 2-4), deteriorated cognition relative to status 90 days earlier, behavioral symptoms that are present a minimum of 4 days per week and not easily altered in the last 7 days (includes wandering, verbal abuse, physical abuse, socially inappropriate or disruptive behavior, and resisting care), receipt of antipsychotic medication or physical restraints in the last 7 days, receipt of antianxiety medication on 3 or more of the last 7 days, receipt of a bedtime sleeping pill on 3 or more of the last 7 days, and indicators of depression or anxiety almost daily in the last 30 days. Some secondary outcomes were altered from their description in the initial protocol and trial registry; this was done while fully blinded (rationale for each change in eAppendix 2 in Supplement 2). RAI-MDS–derived outcomes assess residents at a single time point approximately 135 days after randomization and did not track the resident over the duration of the trial.

Hospital and ED diagnoses were provided by acute care physicians unaware their patient was in a medication timing trial. *International Classification of Diseases, Ninth Revision (ICD-9)* and *International Statistical Classification of Diseases and Related Health Problems, Tenth Revision (ICD-10)* codes defining these outcomes are provided in eAppendix 3 in Supplement 2. The spectrum of reported *ICD-9* and *ICD-10* codes was reviewed by a 3-physician panel (J.E.M.K, R.Y.K., and S.R.G.) to ensure they reflected the intended outcomes, but patient-level outcomes were otherwise accepted without further adjudication.

### Sample Size

BedMed-Frail was conducted using grant funding already in hand to support a different community-based antihypertensive timing trial, BedMed.^38^ Given that even small differences in mortality and major cardiovascular events would be important, our goal was to maximize recruitment and observe as long as available funding permitted. At inception, we anticipated enrolling approximately 1200 participants and had an a priori target of 301 primary outcomes (providing 80% power to detect a 27.6% difference between groups). Midtrial, upon extension of our grant funding deadline to allow longer observation, we raised the expected number of events to 368 (and stated so in the published protocol).

### Interim Analysis

A 5-member independent data safety monitoring board reviewed a single interim analysis of all outcomes on April 11, 2023, at which time 139 primary outcomes had been observed in 481 patients. The board was asked to consider stopping only if there was a *P* ≤ .001 for primary outcome benefit or a *P* ≤ .05 for harm. Following discussion, a recommendation to continue the trial was made. Investigators were fully blinded to all but aggregated data throughout the trial, including the interim analysis, excepting adherence to allocation, knowledge of which was needed to ensure adequate implementation of the intervention. The decision to stop the trial was not influenced by knowledge of interim differences.

### Statistical Analysis

The statistical analysis plan, including predefined regression covariates, has been published (Supplement 1).^25^ Hypothesis testing was 2-sided with a *P* < .05 significance threshold. The primary outcome was analyzed using Cox proportional hazards survival analysis and plotting the cumulative incidence function. Regression covariates, selected by clinicians with experience managing continuing care residents, were felt likely to predict the primary outcome or modulate the intervention’s effects. Baseline characteristics used as covariates were predefined at the protocol stage and all were included in the model including age (<70 years), sex, facility- and level of care–specific mortality rate (prior 3 years), new admission (<60 days), old admission (>3 years), days hospitalized in prior 6 months, Charlson score, facility, total number of blood pressure medications, heart failure, chronic obstructive pulmonary disease, chronic kidney disease, stroke, and diabetes. Age is not a linear predictor of death in long-term care residents; age (<70 years) is meant to capture residents with different disabling conditions (eg, traumatic brain injury and multiple sclerosis). Length of admission was categorical because (1) new admissions (<60 days) can be less medically stable and have higher probability of acute care transfer, and (2) old admissions (>3 years) can be very medically stable and have much longer life expectancy. Other outcomes used Cox proportional hazards or Poisson regression. For time-to-event analyses where mortality was not part of the outcome, Fine-Gray subdistribution hazard models accounted for death as a competing risk. Survival analyses were modified intention-to-treat. There was no missing data, and the proportional hazards assumption was confirmed for all Cox models (eAppendix 2 in Supplement 2). Post hoc, observing a difference between groups, we additionally examined all-cause unplanned hospitalization and ED visits using Poisson regression and including all 379 available events (vs the 235 first occurrences utilized in survival analysis).

Adherence to allocation time denotes the timing of bubble-packed antihypertensive medications 6 months after randomization. For this calculation (percentage of antihypertensive doses administered per allocation) medications dosed more than once daily were considered as a half dose in the morning and a half dose in the evening. Data analysis was conducted from March to August 2024 using SAS version 9.4 (SAS Institute) and R version 4.3.2 (R Project for Statistical Computing).

#### Modified Intension-to-Treat

Our volunteer facilities were fitting the trial in with other initiatives that sometimes took precedence (eg, dealing with the COVID-19 pandemic). For a few facilities, implementation was delayed by approximately 4 months before facility pharmacists acted on their allocation list (eTable 1 in Supplement 2). Given that implementation delays would lessen the ability to detect differences between groups, we switched to modified intension-to-treat for all survival analyses by considering each facility’s randomization date to be the day before the allocation list was actually implemented (determined using pharmacy dispensing data). Individuals in both groups no longer eligible on the date of implementation (ie, those who died, were discharged, stopped all once-daily BP medication, or were hospitalized) were excluded. Full intention-to-treat analyses of all outcomes were conducted as sensitivity analyses.

#### Per-Protocol Analysis

To minimize the effect of nonadherence, we repeated all analyses after excluding all residents (intervention and control) from facilities where, for the bedtime group, less than 60% of 6-month antihypertensive doses were taken per allocation. This was preferred over propensity score matching because excluding poorly performing facilities otherwise maintained the randomization, minimizing the potential for bias.

## Results

### Trial Participants

**Figure 1.**
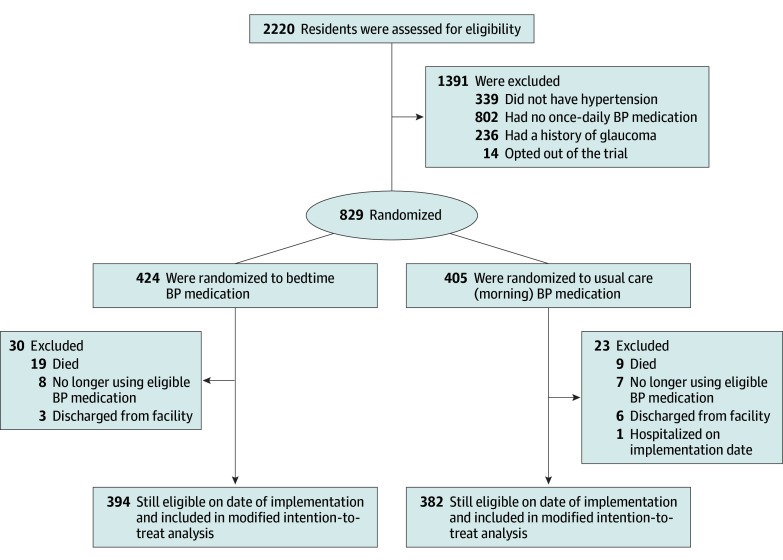
Randomization and Follow-Up in the BedMed-Frail Trial BP indicates blood pressure.

Overall, the 776 analyzed residents (394 bedtime and 382 usual care) had a median (IQR) age of 88 (81-92) years; of these individuals, 562 (72.4%) were female, 367 (47.3%) had diabetes, 307 (39.6%) had coronary artery disease, and 664 (85.6%) had some degree of dementia (Figure 1 and Table 1). Of 843 initially eligible residents, 14 (1.7%) opted-out and 53 (6.3%) became ineligible awaiting implementation. Of all baseline antihypertensive medications, 545 of 591 bedtime group medications (92.2%) and 503 of 565 usual care group medications (89.0%) were once daily, while 46 of 591 bedtime group antihypertensives (7.8%) and 62 of 565 usual care group antihypertensives (11.0%) were dosed 2 times or more daily. Continuing care ward characteristics are in eTable 2 in Supplement 2.

**Table 1.** Baseline Characteristics

Characteristic	Participants, No. (%)	
	Intervention (bedtime) [n = 394]	Control (usual care) [n = 382]
Age, y		
Median (IQR)	88 (80-92)	88 (81-92)
<70	29 (7.4)	34 (8.9)
Sex		
Female	289 (73.4)	273 (71.5)
Male	105 (26.6)	109 (28.5)
Level of care^a^		
DSL4	31 (7.9)	30 (7.9)
DSL4-dementia	18 (4.6)	12 (3.1)
LTC	345 (87.6)	340 (89.0)
Length of stay in facility^b^		
Median (IQR), d	434 (68-955)	357 (67-1095)
<60 d	84 (21.3)	81 (21.2)
60 d to 3 y	224 (56.9)	205 (53.7)
>3 y	86 (21.8)	96 (25.1)
No. of BP medications^c^		
1	235 (59.6)	231 (60.5)
2	124 (31.5)	124 (32.5)
3	32 (8.1)	23 (6.0)
4	3 (0.8)	3 (0.8)
5	0	1 (0.3)
Type of BP medications^d^		
Calcium channel blocker	169 (42.9)	180 (47.1)
Angiotensin-converting enzyme inhibitor	145 (36.8)	148 (38.7)
Angiotensin receptor blockers	116 (29.4)	101 (26.4)
β-Blocker	87 (22.1)	69 (18.1)
Diuretic^e^	73 (18.5)	63 (16.5)
Other types	2 (0.5)	4 (1.0)
No. of non-BP medications, median (IQR)	6 (4-8)	6 (4-8)
Days in hospital, prior 6 mo		
Any	113 (28.7)	99 (25.9)
Total days, mean (SD)	13.6 (29.3)	13.4 (28.7)
ED visit, prior 6 mo		
Any	110 (27.9)	113 (29.6)
No. of ED visits, mean (SD)	0.4 (0.8)	0.4 (0.8)
Charlson score		
Median (IQR)	5 (3-7)	4 (3-6)
0-1	31 (7.9)	41 (10.7)
2-3	102 (25.9)	95 (24.9)
4-5	103 (26.1)	123 (32.2)
≥6	158 (40.1)	123 (32.2)
Comorbidities		
Dementia	338 (85.8)	326 (85.3)
Chronic kidney disease	190 (48.2)	187 (49.0)
Diabetes	196 (49.7)	171 (44.8)
Coronary artery disease	168 (42.6)	139 (36.4)
Heart failure	139 (35.3)	118 (30.9)
Stroke	119 (30.2)	105 (27.5)
Sleep apnea	96 (24.4)	94 (24.6)
Chronic obstructive pulmonary disease	90 (22.8)	79 (20.7)

Abbreviations: BP, blood pressure; DSL4, designated supported living level 4; ED, emergency department; LTC, long-term care.

^a^ The level of care provided at each facility varies. On DSL4 wards, all residents have complex medical needs that require 24-hour on-site presence of a nurse and require lots of assistance with personal care. Some residents may not be ambulatory, and some may not be able to feed themselves. All residents have their medications administered by caregivers. DSL4-dementia wards are the same as DSL4 but add specialized dementia care and precautions to prevent wandering. LTC wards provide the highest level of care. LTC residents require 24-hour availability of a nurse who can respond immediately if needed. Residents may have unpredictable behaviors that put themselves or others at risk. LTC facilities have a higher percentage of registered nurses vs licensed practical nurses providing front-line care and more frequent care plan reassessments.

^b^ New admissions (<60 days) are sometimes less medically stable because they are adjusting to a new care environment and potentially still recovering from an acute health event that led to their loss of independence. In contrast, old admissions (>3 years) are often very medically stable and can have longer life expectancy.

^c^ Of all baseline antihypertensive medications 545 of 591 medications (92.2%) in the bedtime group and 503 of 565 medications (89.0%) in the usual care group were once-daily, compared with 46 of 591 medications (7.8%) in the bedtime group and 62 of 565 medications (11.0%) in the usual care group medications, which were taken more than once a day.

^d^ Combination pills are recorded in the categories of both constituent medications.

^e^ Diuretics were either thiazides, thiazide-like, or potassium-sparing. There were 136 in total and included spironolactone (63 diuretics [46.3%]), hydrochlorothiazide (45 diuretics [33.1%]), indapamide (11 diuretics [8.1%]), chlorthalidone (8 diuretics [5.9%]), a hydrochlorothiazide plus triamterene combination pill (5 diuretics [3.7%]), metolazone (5 diuretics [3.7%]), and amiloride (1 diuretic [0.7%]).

### Follow-Up and Adherence to Allocation

Median (IQR) follow-up was 415 (251-735) days due to high overall all-cause mortality (29.7 per 100 patient-years; median [IQR] time to death, 337 [144-547] days). Loss to follow-up or withdrawal occurred only when residents were discharged from their facility, totaling 14 of 394 participants (3.6%) in the bedtime group and 18 of 382 participants (4.7%) in usual care. At 6 months, the average bedtime group participant used 66.8% of antihypertensive doses in the evening (bedtime or dinnertime) compared with 15.3% of antihypertensive doses in the evening in usual care. Nonadherence occurred largely because some physicians and pharmacists were reluctant to allow the initial switch to bedtime for some patients and some medications, especially diuretics, (eTable 3 in Supplement 2), and not because timing changes were poorly tolerated. Adherence to allocation, assessed monthly over 1 year after randomization, was sustained over time for all antihypertensive medication classes (eFigure 1 and eFigure 2 in Supplement 2).

### Primary Outcome

**Figure 2.**
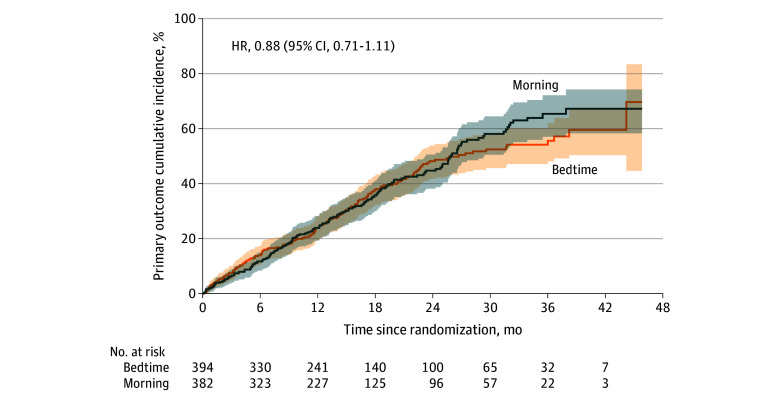
Effect of Medication Timing on Cardiovascular Events and Death Figure shows the cumulative incidence of a first composite primary outcome event (all-cause death or hospitalization/emergency department visit for stroke, acute coronary syndrome, or heart failure) for the comparison of bedtime vs usual care (largely morning) antihypertensive use. HR indicates hazard ratio.

**Figure 3.**
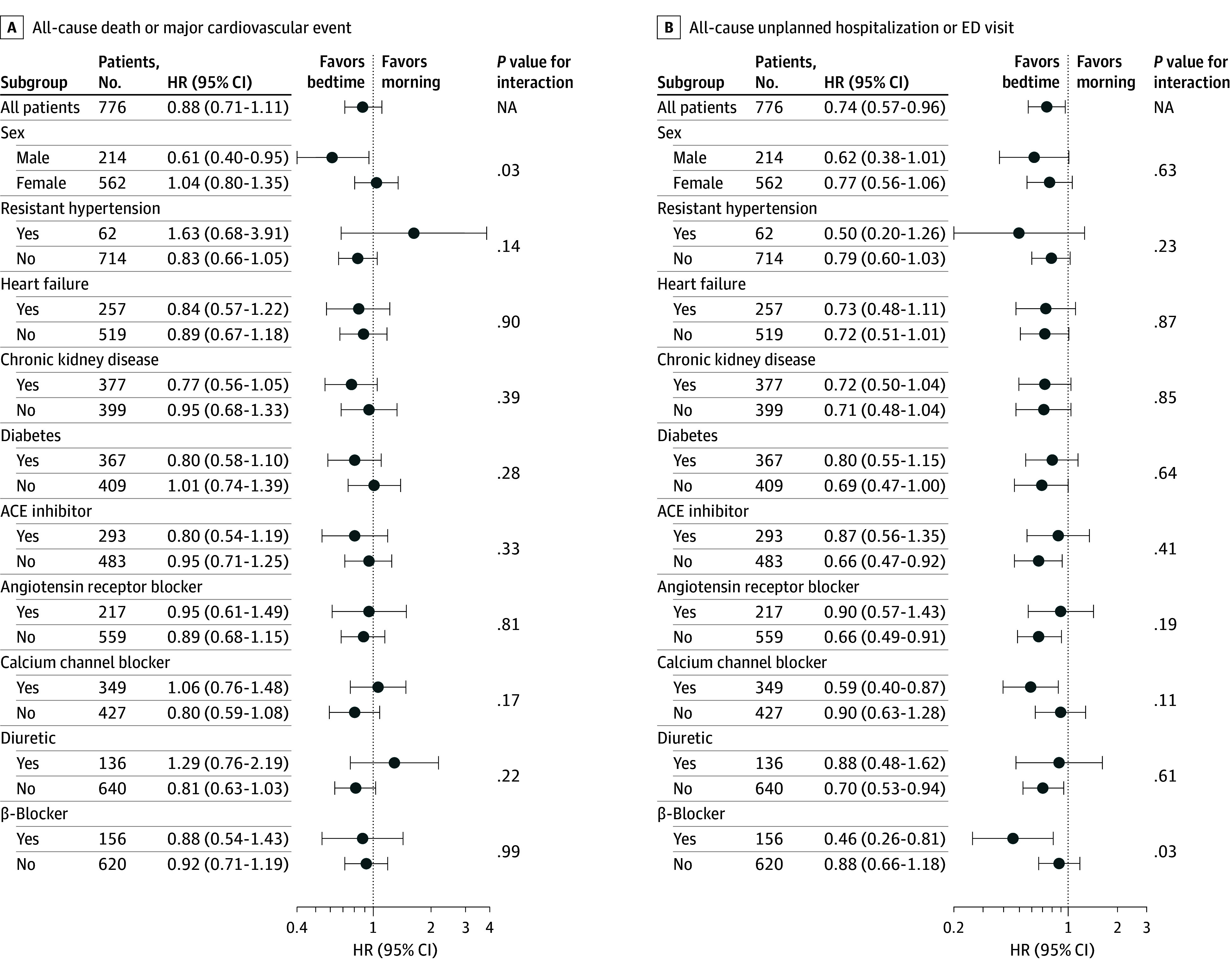
Subgroup Analyses: Primary Outcome and All-Cause Unplanned Hospitalization or Emergency Department (ED) Visit For all-cause death or major cardiovascular event (A) and unplanned hospitalization or ED visit (B), interaction *P* values for the prespecified subgroup analyses shown were obtained from a Cox model employing all covariates preidentified in the statistical analysis plan, and including both the characteristic of interest, and an interaction term between that characteristic and the randomization group. All confidence intervals are adjusted using the same rules for covariate selection as defined in the statistical analysis plan (which fully define order of covariates and number of covariates for inclusion according to the number of events observed). Resistant hypertension was defined as 3 or more antihypertensive medications. ACE indicates angiotensin-converting enzyme; HR, hazard ratio; NA, not applicable.

When exhaustion of funding led the trial to conclude, we had observed 320 primary outcome events (160 in each group), exceeding the 301 events powered for a priori. Of these 320 events, 293 (91.6%) were deaths. Death or major cardiovascular events was not different between groups (Figure 2), with 29.4 events per 100 patient-years in the bedtime group and 31.5 events per 100 patient-years in usual-care (adjusted hazard ratio [aHR], 0.88; 95% CI, 0.71-1.11; *P* = .28; unadjusted HR, 0.93; 95% CI, 0.75-1.16). The same was true for all individual components of the primary outcome (Table 2), and all prespecified subgroups (Figure 3) save sex, where event rates were lower with bedtime administration in males (aHR, 0.61; 95% CI, 0.40-0.95; *P* = .03) and no different in females (aHR, 1.04; 95% CI, 0.80-1.35; *P* = .79). However, given 10 subgroups (ie, multiple hypothesis testing), much lower *P* values would be needed for statistical significance (*P* < .005 if Bonferroni corrected).

**Table 2.** Primary and Secondary Outcomes—Main Analysis

Outcome	Treatment (bedtime) [n = 394]		Control (usual care) [n = 382]		Adjusted RR (95% CI)
	Participants, No. (%)	Rate/100 patient-y	Participants, No. (%)	Rate/100 patient-y	
Primary: major adverse cardiovascular event	160 (40.6)	29.4	160 (41.9)	31.5	0.88 (0.71-1.11)^a^
Secondary: efficacy					
Primary outcome components					
All-cause mortality	157 (39.8)	28.7	157 (41.1)	30.7	0.89 (0.71-1.11)^a^
Hospitalization for stroke	3 (0.8)	0.5	7 (1.8)	1.4	0.40 (0.10-1.57)^a^
Hospitalization for myocardial infarction or acute coronary syndrome	2 (0.5)	0.4	2 (0.5)	0.4	0.93 (0.13-6.51)^a^
Hospitalization for heart failure	8 (2.0)	1.5	6 (1.6)	1.2	1.26 (0.44-3.63)^a^
All-cause unplanned hospitalization or emergency department visit	107 (27.2)	22.6	128 (33.5)	30.0	0.74 (0.57-0.96)^a^
Secondary: safety^b^					
Falls or fractures					
Nonvertebral fracture	9 (2.3)	1.7	10 (2.6)	2.0	0.84 (0.34-2.07)^a^
Fall in the past 30 d	53 (15.4)	NA	54 (15.9)	NA	0.97 (0.67-1.42)
Cognitive or behavioral					
Deteriorated cognition^c^	32 (9.3)	NA	35 (10.3)	NA	0.92 (0.57-1.48)
Problem behaviors^d^	50 (14.5)	NA	42 (12.4)	NA	1.13 (0.75-1.71)
Use of antipsychotic medication or physical restraints^e^	64 (18.6)	NA	84 (24.7)	NA	0.74 (0.53-1.03)
Indicators of depression or anxiety almost daily^f^	56 (16.2)	NA	48 (14.1)	NA	1.11 (0.75-1.64)
Use of antianxiety medication^g^	25 (7.2)	NA	23 (6.8)	NA	1.06 (0.60-1.87)
Use of bedtime sleeping pill^g^	30 (8.7)	NA	27 (7.9)	NA	1.11 (0.66-1.87)
Other					
Partial or full thickness skin ulceration (stage 2-4)	31 (9.0)	NA	37 (10.9)	NA	0.83 (0.51-1.33)
Urinary incontinence^h^	302 (87.5)	NA	287 (84.4)	NA	1.04 (0.88-1.22)

Abbreviations: HR, hazard ratio; NA, not applicable; RR, risk ratio.

^a^ Risk for the primary outcome, secondary efficacy outcomes, and nonvertebral fractures was measured using hazard ratios with 95% CIs.

^b^ Except for nonvertebral fracture, all safety outcomes are as recorded by nurses on the Resident Assessment Instrument Minimum Data Set 2.0 assessment closest to day 135 after randomization.

^c^ Deteriorated cognition relative to status 90 days earlier (qualitative global assessment by nurse providing direct care).

^d^ Behavioral symptoms that are present a minimum of 4 days per week and not easily altered in the last 7 days (includes wandering, verbal abuse, physical abuse, socially inappropriate or disruptive behavior, and resisting care).

^e^ In the last 7 days (includes chair, trunk, or limb restraints, but does not include bedrails).

^f^ In the last 30 days. Applies to 16 discrete mood and anxiety related observations of the resident, any one of which being listed as present 6 or more days per week would qualify.

^g^ On 3 or more of the last 7 days.

^h^ Two or more times per week.

### Sensitivity Analyses

#### Per-Protocol Analysis and Full Intention-to-Treat Analysis

For the 13 of 17 residences dispensing 60% or more of bedtime group antihypertensive doses per allocation (which includes 559 of 776 residents [72.0%]), at 6 months, the average bedtime group participant used 78.8% of antihypertensive doses in the evening compared with 14.8% of antihypertensive doses in the evening in usual care. Repeating the primary outcome analysis in this population (eTable 4 in Supplement 2), using an identical model, provided near identical results (aHR, 0.88; 95% CI, 0.67-1.15; *P* = .35) (eTable 5 in Supplement 2). Repeating the primary outcome analysis (identical model) in the intention-to-treat population (eTable 6 in Supplement 2) also produced similar results (aHR, 0.91; 95% CI, 0.74-1.13; *P* = .39) (eTable 7 in Supplement 2).

### Secondary and Safety Outcomes

Secondary efficacy and safety outcomes were no different between groups (Table 2), excepting all-cause unplanned hospitalization and ED visits which favored the bedtime group (22.6 events per 100 patient-years in the bedtime group vs 30.0 events per 100 patient-years in the control group; aHR 0.74; 95% CI, 0.57-0.96; *P* = .02). However, significance was lost in a post hoc Poisson regression utilizing all 379 available events, with 34.8 events per 100 patient-years in the bedtime group and 36.9 events per 100 patient-years in usual care (adjusted relative risk, 0.87; 95% CI, 0.71-1.07; *P* = .20) (eTable 8 in Supplement 2). Unadjusted HRs for secondary outcomes were not materially different (eTable 9 in the Supplement). All-cause death over time is provided in eFigure 3 in Supplement 2.

## Discussion

This RCT found that among frail older adults residing in Alberta nursing homes, those switching once-daily antihypertensive medications to bedtime had similar rates of all-cause death, stroke, acute coronary syndrome, or heart failure as those continuing largely morning antihypertensive use. The same was true for the hypothesized adverse effects of falls and fractures, decubitus ulcers, and worsening cognition or behavioral problems.

Our findings agree with Mackenzie et al,^9^ a general population RCT of antihypertensive timing reporting neither benefit nor harm for bedtime vs morning antihypertensive use, and they extend the Mackenzie et al^9^ findings to a seldom studied population for whom risks and benefits could have differed. We similarly agree with a cohort study carried out by the Spanish Ambulatory Blood Pressure Monitoring Registry,^10^ who followed 35 129 morning and 6723 evening antihypertensive users and reported no difference in all-cause or cardiovascular death in an adjusted analysis. Our findings also augment the Cochrane review on this topic,^39^ which had too few events to draw conclusions on cardiovascular outcomes as a result of excluding trials where participants had more than 1 antihypertensive. Neither did the Cochrane review report on specific adverse effects. In contrast, our findings differ markedly from the 2 studies by Hermida et al^7,8^ reporting bedtime antihypertensives to convey 61% and 45% reductions in major adverse cardiovascular events , and 49% and 45% reductions in all-cause mortality, respectively. While differences in trial design might explain some of this discrepancy, antihypertensive medications, and their administration time, might also have less influence on the major causes of mortality and morbidity in frail end-of-life populations, limiting the ability of any BP-related intervention to show benefit.

Although we report no difference in a composite of death or major cardiovascular events, 293 of 320 primary outcome events (91.6%) were all-cause deaths. Hence, while our intervention can be said to have had little effect on all-cause death, the effect on major cardiovascular events is unclear and some might consider our point estimate (HR, 0.88) to be clinically important, were it statistically significant. We also found a secondary outcome, all-cause unplanned hospitalization and ED visits, to be less frequent in the bedtime group. Given that we had 16 primary and secondary outcomes, it would not be surprising for one outcome to be statistically different at *P* < .05 by chance alone, and a post hoc Poisson regression utilizing all 379 available events found no difference between groups, suggesting this finding might be spurious. However, it is also conceivable that conditions and problems we did not examine were influenced by antihypertensive timing and led to the difference in acute care presentations.

### Limitations

Our findings are limited in generalizability outside of continuing care settings, and our RAI-MDS–derived outcomes only examined the state of the resident approximately 135 days after randomization (ie, falling concerns, skin ulcers, and behavioral/cognitive problems outside this cross-section of time would not be detected). They are also limited by potentially less aggressive management of cardiovascular events in nursing home residents (which might reduce the rate of acute care transfer) and by the open label nature of our trial, although mortality is objective, and acute care physicians providing hospitalization and ED diagnoses would have been unaware their patient was in a clinical trial. We were also limited by nonadherence to the timing intervention, largely due to physician and pharmacist reluctance to approve the initial switch to bedtime for some patients, and some medications (eg, diuretics), plus the inclusion of twice-daily antihypertensives. However, our per-protocol analysis (72.0% of participants), which had near identical results to the primary analysis (and which preserved the randomization by including all residents of the included wards), was carried out on high-performing wards where 78.8% of bedtime group antihypertensive doses were taken in the evening at 6 months, compared with only 14.8% evening dosing in usual care control. We had complete capture of prescribing data; these medications were bubble-packed and nurse-administered, and adherence rates were stable over time. Our findings are simultaneously strengthened by only 1.7% of otherwise eligible residents opting out, and by a low 4.1% combined dropout and loss to follow-up.

## Conclusions

In this RCT of antihypertensive timing in frail older adults, we found bedtime vs usual care (largely morning) administration of antihypertensive medications to provide neither benefit (in terms of an all-cause mortality–dominated composite outcome), nor harm (in terms of falls or fractures, decubitus ulcers, and worsening cognition or behavioral problems). These findings suggest that for frail older adults, administration time likely has little influence on the benefits and risks of antihypertensive medications.

## Notes

Supplement 1.
Trial Protocol and Statistical Analysis Plan

Supplement 2. **eAppendix 1.** BedMed-Frail Committees, Investigators, and Staff **eAppendix 2.** Definitions, Appropriateness of the Statistical Analyses, and Changes to Secondary Outcomes **eAppendix 3.** Electronic Health Data (*ICD-9/ICD-10*) Outcome Definitions **eTable 1.** Adapted Randomization Dates **eTable 2.** Continuing Care Ward Characteristics **eTable 3.** Percentage of Antihypertensive Medication Doses at Bedtime for Individual Medications by Class of Antihypertensive (Baseline and 6-Months) **eFigure 1.** Mean % of Antihypertensive “Doses” in the Evening (Primary Analysis Participants) **eFigure 2.** Mean % of Antihypertensive Doses in the Evening (Per-Protocol Participants) **eTable 4.** Baseline Patient Characteristics Comparing Residents Included vs Excluded From Per-Protocol Analysis (Based on Exclusion of Facilities With < 60% Adherence) **eTable 5.** Primary and Secondary Outcomes—Per-Protocol Analysis **eTable 6.** Baseline Patient Characteristics Comparing Residents Included vs Excluded From Full Intention-to-Treat Analysis **eTable 7.** Primary and Secondary Outcomes—Full Intention-to-Treat Analysis **eTable 8.** Analysis of Unplanned All-cause Hospitalization/ED Visits as a Continuous Variable (Poisson Regression), Counting All Events During Follow-Up Period **eTable 9.** Unadjusted Hazard Ratios for Primary and Secondary Outcomes **eFigure 3.** Effect of Medication Timing on All-Cause Death

Supplement 3.
Data Sharing Statement
