# Supplementary material for: Bedtime vs Morning Antihypertensive Medications in Frail Older Adults: The BedMed-Frail Randomized Clinical Trial
Source: JAMA Netw Open. 2025 May 12;8(5):e2513812. doi: 10.1001/jamanetworkopen.2025.13812 (PMC12070236; doi:10.1001/jamanetworkopen.2025.13812)
Supplement: Supplement 1. — Trial Protocol and Statistical Analysis Plan [file jamanetwopen-e2513812-s001.pdf]

TABLE OF CONTENTS

This supplement contains the following items:

1. BedMed-Frail Protocol (version 0.03 dated Nov 5, 2019), as submitted to the University of Alberta's Health Research Ethics Board
2. Log of all protocol amendments

*Note: The final version of the BedMed-Frail protocol, along with the final analysis plan, was published on Aug 1, 2023 as: *Garrison SR, Youngson E, Perry DA, Campbell FN, Kolber MR, Korownyk C, Allan GM, Green L, Bakal J. Bedtime versus morning use of antihypertensives in frail continuing care residents (BedMed-Frail): protocol for a prospective, randomised, open-label, blinded end-point pragmatic trial. BMJ Open. 2023 Aug 1;13(8):e074777. doi: 10.1136/bmjopen-2023-074777. PMID: 37527898; PMCID: PMC10394547.*

BedMed-Frail: Does the potential benefit of bedtime antihypertensive prescribing extend to frail populations?

OVERVIEW: The BedMed trial (led out of the U of A and funded by both Alberta Innovates Health Solutions, and the Canadian Institutes for Health Research) is a pragmatic multi-provincial trial intended to determine whether bedtime antihypertensive use, as compared to conventional morning use, reduces major adverse cardiovascular events in community dwelling primary care patients. BedMed-Frail, led by the same group of investigators, is a complementary but separate randomized trial evaluating whether the risks and benefits of bedtime antihypertensive use differ in a long-term care (LTC) population. To accomplish this, BedMed-Frail randomizes residents in participating Alberta LTC facilities to the antihypertensive medication timing intervention (i.e. bedtime versus conventional morning use) and draws both trial outcomes and baseline covariates from routinely collected electronic health data – using both administrative claims data and the Resident Assessment Instrument Minimum Data Set (RAI-MDS).

PURPOSE: To determine the optimal time of day for blood pressure lowering medication to be administered to long-term care residents.

HYPOTHESIS: Bedtime administration will result in fewer adverse health events.

JUSTIFICATION:

A) THE BURDEN OF HYPERTENSION: In Canada cardiovascular disease (CVD), most notably stroke, heart attack (MI), and congestive heart failure (CHF) accounts for 1 in 3 deaths, and 1 in 6 hospitalizations.[1] Hypertension (high blood pressure), a modifiable risk factor present in 1 of 5 Canadian adults, is the leading cause of stroke and predisposes patients to heart attack, congestive heart failure, kidney disease and dementia.[2] Using population-based Canadian administrative data (2002 - 2010), it was recently estimated that Canadian healthcare costs attributable to hypertension were $13.9 billion in 2010 and projected to rise to $20.5 billion in 2020.[3] If high blood pressure (BP) can be reduced in a way that is more effective at preventing these life threatening and potentially disabling conditions, better health outcomes and substantial cost savings are possible. One way to improve the effectiveness of BP medications may be to administer them at bedtime.

B) THE OPPORTUNITY BEDTIME PRESCRIBING MIGHT PROVIDE (the "MAPEC" trial): Blood pressure (BP) normally exhibits a circadian rhythm with relatively lower pressures during sleep.[4] Lack of this sleep time "dip" correlates strongly with adverse cardiovascular events and BP correlates most strongly with such events when measured at night (i.e. during sleep).[5-8] Motivated by such observations, Spanish researchers studied the effect of taking BP medication at BEDTIME (when the effect on nighttime BP would be greatest) versus conventional morning use, when most BP medications (meds) are taken. The results of this study (the MAPEC trial) were striking.[9] Over a median 5.6 years follow-up, adverse cardiovascular events occurred in 187 of 1084 subjects taking BP medication in the morning but only 68 of 1072 subjects who took their BP medication at bedtime (relative risk 0.39, 95%CI [0.29-0.51], $p < 0.001$). This *61% reduction* in adverse events was similar for all individual components of the primary outcome (including death from all causes, stroke, MI, new angina pectoris, CHF and retinal artery occlusions). If true, a switch to bedtime prescribing would have more impact on the health of hypertensive Canadians than whether high BP is treated at all. And yet this trial failed to change practice. This is because *replication of such surprising results is required for these findings to be believed* – primarily because the benefit seems "too good to be true", far exceeding

all other methods of CVD risk reduction (e.g. high dose statins convey a 35% relative risk reduction, morning BP meds 30%, exercise 25%, aspirin 15%).[10]

C) OTHER STUDIES IN HUMANS: Neither Oct 2013 literature review,[11] 2011 Cochrane systematic review,[12] nor our more current (2018) search of the literature, found any RCTs (besides MAPEC) evaluating the effect of BP medication timing on mortality/morbidity.[11,12] Other trials look only at 1) how medication timing affects BP (not cardiovascular events), or 2) compare AM vs. bedtime use of differing drug classes. ***1) Effect on BP:*** Clearly, except for dihydropyridine calcium channel blockers such as amlodipine whose BP lowering is largely independent of administration time, medications from every antihypertensive class (when taken at bedtime instead of AM) *will preferentially lower overnight BP*.[13,14] ***2) Comparing AM vs. bedtime use of different drug classes:*** A 2014 review identifies 5 such RCTs and attempts to combine them in meta-analysis.[15] Although the authors report overall benefit to bedtime prescribing it is difficult to see how such disparate trials could be meaningfully combined. Two of these studies also found no difference - although one (FACET) was a trial of morning fosinopril versus evening amlodipine[16] (which doesn't preferentially lower overnight BP) and the other (CONVINCE) was a trial of morning BP meds (atenolol or HCTZ) vs. evening controlled onset delayed release verapamil[17], which releases upon waking each morning (i.e. it was NOT a trial of overnight BP lowering).

D) ANIMAL STUDIES: In an examination of 12 mouse organs, 43% of all protein coding genes showed circadian rhythms in transcription[18] - with transcription "rush hours" typically preceding dawn or dusk. This includes genes such as VEGF (vascular endothelial growth factor) that regulate angiogenesis. Conceivably, elevated BP at night and elevated BP during the day may have very different metabolic effects on a variety of tissues. Altering the time at which peak BP lowering occurs could potentially alter the benefit conveyed.

E) RESEARCH IN PROGRESS: Four research teams have taken steps in this area: 1) The National Heart Lung and Blood Institute (USA) funded a 2013 UH2 planning grant for a trial looking to confirm MAPEC. Unfortunately no further funding was announced and *the intended trial was never registered*.[11] 2) Ramon Hermida (MAPEC lead author) is pursuing a second study (HYGIA)[19] in which antihypertensive treatment time is randomized and mortality and morbidity examined (this time randomizing ALL BP meds) however, *an investigator cannot independently reproduce their own work* - especially results as extraordinary as MAPEC reports. 3) The British Heart Institute is funding a BP medication timing trial (TIME)[20] looking at patient oriented outcomes (e.g. stroke, MI, CHF). This trial is ongoing and expects mature data in roughly 2 years (estimate from e-mail communication with TIME investigative team). However this trial interacts with subjects exclusively via website questionnaire and e-mail - which biases towards a healthier, younger population. 4) Alberta Innovates Health Solutions and the Canadian Institutes of Health Research (CIHR) are funding the BedMed Initiative, an RCT in community dwelling hypertensive patients that is being conducted in multiple Canadian provinces and led out of the U of A. This trial is currently enrolling (roughly 1500 participants randomized) and expects mature data in 2021.

F) THE NEED FOR EVIDENCE IN FRAIL OLDER ADULTS: In a systematic sampling of RCTs published in high impact journals, 38.5% of RCTs excluded older adults, 81.3% excluded individuals with common medical conditions, and 54.1% excluded individuals receiving commonly prescribed medications.[21] As a result, the vast majority of RCT evidence does not apply to frail older adults. This is doubly true for long-term care residents, in whom cognitive impairment / inability to consent further hinders interventions being studied in such populations despite their unique health status and goals of care.

Given the risk/benefit of all interventions, including the timing of medications, could differ meaningfully in such highly frail populations, the BedMed investigators believe evidence for such frail populations should be sought when it is ethical to do so.

OBJECTIVES:
A) To determine if switching ≥ 1 BP Med to bedtime alters cardiovascular outcomes.
B) To determine if switching ≥ 1 BP Med to bedtime alters behavior outcomes.
C) To determine differences in acute care costs between morning and bedtime users.

RESEARCH METHOD/PROCEDURES:

A) SETTING: Alberta is home to the Institute for Continuing Care Education and Research (ICCER), a network of long-term care facilities intent on providing new knowledge relevant to improving quality of care for their residents. Working with their Executive Director (Sandra Woodhead Lyons) the BedMed investigators have approached, and obtained, support for BedMed-Frail from multiple ICCER-affiliated LTC organizations. Currently this includes the Bethany Care Society, Capital Care, Shepherd's Care, Sherwood Care, Wingkei, and Lifestyle Options Retirement Communities. Other facilities might join in before randomization begins. Collectively these facilities would have approximately 1200 eligible residents during the 2-3 year study period.

B) DATA SOURCE*:* All our outcomes and baseline covariates are derived from routinely collected electronic health data. This includes Ministry of Health / Health Authority administrative claims data (physician billings, vital statistics, emergency and hospital separations, prescriptions dispensed) and quarterly clinical care data captured by all LTC facilities in the Minimum Data Set (MDS) which is recorded quarterly in all institutions for all residents. To access and analyse this data (both administrative claims and MDS) we are partnering with the Alberta SPOR Data Platform through Jeff Bakal (*Senior Biostatistician, Patient Health Outcomes and Clinical Effectiveness Unit, Alberta Health Services*) and his team of AHS biostatisticians.

C) BEDMED-Frail TRIAL PROTOCOL:

Design: Event-driven Prospective Randomized Open Blinded End-Point (PROBE)[22] Randomized Trial.

Population: Hypertensive long-term care (LTC) residents using BP lowering medication.

<u>Inclusion Criteria</u>: 1) Hypertension diagnosis as indicated by ≥ 2 such billing diagnoses at any time in the administrative claims data by any provider; 2) ≥ 1 ONCE DAILY BP lowering medication; 3) LTC resident in a participating facility.

<u>Exclusion Criteria</u>: 1) Personal history of glaucoma or use of glaucoma medications. *Glaucoma is an exclusion because nocturnal hypotension (i.e. excessively low blood pressure while sleeping) has been associated with ischemic optic neuropathy in such patients.*[23-26] 2) Any family member or treating physician (all of whom will be notified of the LTC facilities participation in this initiative) requesting the patient not participate.

Intervention: Switching (*one at a time as tolerated*) one or more morning BP meds to BEDTIME (or *maintaining* bedtime BP meds if already taken at that time). The timing of loop diuretics (which can cause meaningful diuresis), and BP medications taken more than once a day, will not be altered. If more than one medication change is needed, each change will take place one week apart.

Comparator: Maintaining (or switching to) morning BP medication use.

Outcomes: Outcomes are drawn from administrative claims data and quarterly MDS reporting. The Alberta SPOR Data Platform will monitor our primary outcomes *quarterly* during the trial, and the trial will conclude once 301 participants have experienced primary outcome events. Outcomes include:

- *Primary:* Composite of all-cause death and hospital admission or emergency room visit for acute coronary syndrome/MI, heart failure, or stroke (using administrative data based case definitions with validated coding algorithms[27-29]).
- *Secondary:* 1) Each element of the primary outcome alone; 2) All-cause hospitalization; 3) Acute Care Costs (from each admissions resource intensity weight & length of stay).

The following additional outcomes (unless otherwise indicated) will be determined from the first available ***MDS Quarterly assessment*** *in the 3 - 6 month window post randomization:*

- *Safety*: 1) Fell (past 30 days); 2) Any Fracture (from administrative claims data over the study duration – not MDS data); 3) Full Thickness Skin Ulceration (stage 3 or 4).
- *Tolerability:* Bladder Continence.
- *Process:* 1) % of once daily BP medications taken according to allocation (via hand audit of medication records 6 months post date of randomization); 2) Number of once daily BP medications 6 months post date of randomization (via administrative claims data). 3) Number of BP medications taken more than once daily.
- *Exploratory (Cognitive / Behavioral):* 1) Deteriorated cognition relative to status 90 days prior; 2) Receipt (last 7 days) of a) Antipsychotic Medication, b) Antianxiety Medication, c) Hypnotic Medication, d) Physical Restraints; 3) Behavioral symptoms that are present a minimum of 4 days per week and not easily altered including a) Wandering, b) Verbal Abuse, c) Physical Abuse, d) Socially Inappropriate or Disruptive Behaviour, e) Resisting Care; 4) Indicators of Depression or Anxiety Almost Daily (last 30 days).

Baseline Data:

- Baseline annualized rate of the primary outcome in the resident's facility (last 3 years)
- Age
- Gender
- Location (Calgary / Edmonton / Other)
- Level of Care Designation (Long Term Care, Long Term Care-Dementia, Supportive Living, Supportive Living-Dementia, Personal Supportive Living)
- Length of stay in LTC facility
  - New admission (last 60 days)
  - Old Admission (≥ 3 years)
- Charlson score

- Key Comorbidities - requiring 2 claims or 1 in-hospital diagnosis (any Dx, not just primary Dx) over the duration of available administrative claims:
    - Diabetes
    - Coronary Artery Disease (CAD)
    - Stroke
    - Osteoarthritis (OA)
    - Congestive Heart Failure (CHF)
    - Chronic Obstructive Pulmonary Disease (COPD)
    - Hip Fracture – for this characteristic only 1 diagnosis is required
    - Chronic Kidney Disease (CKD) – for this characteristic only 1 diagnosis is required (since family physicians rarely have CKD dedicated visits), or 2 GFR < 40
    - Dementia
    - ≥ 2 of the above Key comorbidities
- TIA (1 claim or 1 in-hospital Dx)
- Stroke or TIA (1 claim or 1 in-hospital Dx)
- Dialysis (1 procedure)
- Total number of medications
- Total number of BP medications
- Days in hospital in prior 6 months
- Number of ER visits in prior 6 months
- *Collected for the purpose of inclusion / exclusion*
    - *Hypertension (2 claims or 1 in-hospital) – inclusion criteria*
    - *Any once daily hypertension medication – inclusion criteria*
    - *Glaucoma – exclusion*

D) Power/Sample Size Estimate:

<u>Expected Recruitment</u>: At the time of this application we are collaborating with multiple ICCER-affiliated LTC organizations that wish to participate in this initiative. Most of these organizations have multiple facilities, many of which have multiple buildings or wings. Using Canadian Primary Care Sentinel Surveillance (CPCSSN) EMR data (accessed for the purpose of this application) we know that 53% of Canadians 80 yrs and older are listed as currently taking daily BP lowering medication. In our experience most of these medications are maintained upon nursing home admission. This suggests, given the 18-month median lifespan of newly admitted patients (and 9 month median lifespan of those already resident at the time of the study), that we should easily obtain 1200 residents total over the roughly 2-3-year duration of the trial.

<u>Power:</u> Utilizing well described methods for sample size determination, inflating the number of events to account for noncompliance, conservatively setting the median lifespan at 3 years to account for the inclusion of supportive living residents who, on average, live longer than the expected 18 months in long-term care facilities and assuming 33% of patients will be censored prior to any events, we find that 1232 patients would have 80% power (at a p of 0.05) to detect a difference between groups in the primary outcome of 27.6%. Although smaller differences would still be clinically meaningful, this difference is less than the 55% relative reduction in total mortality claimed in the MAPEC trial that we are trying to reproduce, and it is a very conservative minimum estimate for the actual power that the study will have. Additional power, while not directly calculable, will come in 2 ways: 1) These calculations assume no meaningful covariates and we have several covariates that are likely to be predictive of each outcome – in particular the resident's

level of care designation, and 2) we will continue to seek new facilities to partner with over the course of the trial – which should increase the number of residents available.

<u>Overall</u>: We expect to randomize a minimum of 1200 residents and observe over a roughly 2-3 year period until 301 primary outcomes have occurred (with those outcomes being reported to us quarterly by the Alberta SPOR Data Platform). At a minimum this will provide sufficient power (80% power to detect a 27.6% difference) to validate or refute the MAPEC trials claim of a 55% reduction in mortality, and will likely provide substantial additional fidelity to detect smaller differences that are not currently calculable (as a result of meaningful covariates, continuous outcomes, and the expectation of additional facilities joining the project in coming months).

E) Confirmation of data accuracy: Prior to randomization the data steward will produce a list of all residents they believe to currently reside at each of the participating facilities. They will simultaneously request a list of all admitted residents from each participating facility and will compare these two lists to ensure there are no errors in identifying residents.

F) Recruitment / Patient Flow: *(Flow Diagram - appendix pg. 6)*

1. *<u>Informing Patients / Families / Primary Care Providers</u>:* Prior to facilities being randomized, we will send letters to all next of kin, and all attending primary care providers, notifying them that this study is taking place and that blood pressure medications might have their timing switched to bedtime. If family or physicians object, we will ask them to e-mail or call a toll free number to let us know the resident's name and the facility they reside in and we will exclude them from the timing change. A number will also be provided to speak with a BedMed physician if families or physicians have questions about the study.
2. *<u>Randomization</u>:* A minimum of one month after the last notification letter is mailed, eligible residents who have not opted out of participation will be randomized using random blocks of 2 or 4 with no stratification.
3. *<u>Implementing the Intervention</u>:* Each facility's pharmacist will be in charge of implementing the intervention. The pharmacy will receive a list of eligible residents directly from the AHS biostatistician supporting the study using an e-mail with a secure link. Only once a day BP medications will be changed and the recommendation is to change BP medications in the order ABCD-O (Angiotensin Inhibitor / Angiotensin Receptor Blocker, Beta-Blocker, Calcium Channel Blocker, Diuretic, Other). If there are multiple once daily BP medications, only one timing change will be made per week. If the pharmacist is aware of any clinical reason why a particular timing would be better they will follow their clinical judgment. Patients with known glaucoma, or who use glaucoma medications, will keep to morning BP medications. A BedMed physician will be available 24 hours for pharmacist questions related to timing changes.
4. *<u>Study Follow-up</u>:* Post implementation of the intervention, the study team will monitor administrative claims and RAI-MDS data quarterly. The study will end once 301 primary outcome events have been observed.

PLAN FOR DATA ANALYSIS:

Interim Analysis: An independent data safety monitoring board (DSMB) organized and chaired by Jim Wright (Cochrane Hypertension Review Group Coordinating Editor) will review all data upon reaching 150 events (~18 months in). If p is ≤ 0.001 for benefit (the

Haybittle-Peto boundary – recommended to reduce the chance of stopping too early and magnifying benefit),[30] or if p is ≤ 0.05 for harm, the DSMB will apply clinical judgement and make recommendations to the steering committee on whether the trial should break early.

Statistical Analysis: The primary outcome will be analyzed by a Proportional Hazards Survival Analysis using multiple patient level covariates including: Age <70, Gender, Location (Edmonton / Calgary / Other), New admission (< 60 days on date of randomization), Old admission (≥ 3 years on date of randomization), Level of Care Designation (i.e. LTC, LTC-D, SL-D, SL, PSL), Days hospitalized in the prior 6 months, Number of ER visits in the prior 6 months, Charlson score, Total number of medications, Total number of BP medications, Dementia. Our AHS data team will be connecting with facilities approximately once per month to determine level of care designation (i.e LTC, LTC-D, SL, SL-D) on each new resident who is eligible to participate in BEDMED-Frail. The purpose of this data is to allow for adjustment of this variable, listed above.

THE CASE FOR RANDOMIZATION WITH A WAIVER OF CONSENT: As important as this work is, it is not feasible without a waiver of consent and it is important to clearly outline to the ethics board why we believe such a waiver is warranted.

1. The only change any patient will make in this trial is to a medication timing with evidence for greater safety (i.e. timing will not change for control institutions).
2. Patients and their families are not currently consulted about what time of day their medications are administered.
3. Current practice is to generally ignore the time of day patients took their blood pressure medication pre-admission and to administer those pills at an arbitrary time of the facilities choosing (typically morning for once daily medication).
4. Patients/families/primary care providers will be notified of the change in policy and given the opportunity to exempt their loved one / patient from participating.
5. After the initial change, patients and providers are free to change back to morning administration for any reason without the need for justification.
6. The vast majority of nursing home patients are unable to provide their own consent as a result of dementia, and family members are likely unable to provide that consent for them as a result of legislation.
7. If a waiver of consent is not possible the trial cannot go forward and the unique information to guide and optimize the care of such patients may never be obtained.

References

1. S. Dai CB, A. Bienek, P. Walsh, P. Stewart, A. Wielgosz, Public Health Agency of Canada,. *2009 Tracking Heart Disease and Stroke in Canada*. Report from the Canadian Chronic Disease Surveillance System.http://www.phac-aspc.gc.ca/publicat/2009/cvd-avc/index-eng.php. Accessed March 18, 2015.

2. Public Health Agency of Canada. Hypertension in Canada 2010. Report from the Canadian Chronic Disease Surveillance System.http://www.phac-aspc.gc.ca/cd-mc/cvd-mcv/ccdss-snsmc-2010/pdf/CCDSS_HTN_Report_FINAL_EN_20100513.pdf Accessed Sept 10 2015.

3. Weaver CG, Clement FM, Campbell NR, et al. Healthcare Costs Attributable to Hypertension: Canadian Population-Based Cohort Study. *Hypertension.* 2015;66(3):502-508.

4. Veerman DP, Imholz BP, Wieling W, Wesseling KH, van Montfrans GA. Circadian profile of systemic hemodynamics. *Hypertension.* 1995;26(1):55-59.

5. Clement DL, De Buyzere ML, De Bacquer DA, et al. Prognostic value of ambulatory blood-pressure recordings in patients with treated hypertension. *N Engl J Med.* 2003;348(24):2407-2415.

6. Verdecchia P, Porcellati C, Schillaci G, et al. Ambulatory blood pressure. An independent predictor of prognosis in essential hypertension. *Hypertension.* 1994;24(6):793-801.

7. Ben-Dov IZ, Kark JD, Ben-Ishay D, Mekler J, Ben-Arie L, Bursztyn M. Predictors of all-cause mortality in clinical ambulatory monitoring: unique aspects of blood pressure during sleep. *Hypertension.* 2007;49(6):1235-1241.

8. Fagard RH, Celis H, Thijs L, et al. Daytime and nighttime blood pressure as predictors of death and cause-specific cardiovascular events in hypertension. *Hypertension.* 2008;51(1):55-61.

9. Hermida RC, Ayala DE, Mojon A, Fernandez JR. Influence of circadian time of hypertension treatment on cardiovascular risk: results of the MAPEC study. *Chronobiol Int.* 2010;27(8):1629-1651.

10. Best Science Medicine. The Absolute CVD Risk/Benefit Calculator. http://chd.bestsciencemedicine.com/calc2.html Accessed Aug 31 2015.

11. Carter BL, Chrischilles EA, Rosenthal G, Gryzlak BM, Eisenstein EL, Vander Weg MW. Efficacy and safety of nighttime dosing of antihypertensives: review of the literature and design of a pragmatic clinical trial. *J Clin Hypertens (Greenwich).* 2014;16(2):115-121.

12. Zhao P, Xu P, Wan C, Wang Z. Evening versus morning dosing regimen drug therapy for hypertension. *Cochrane Database Syst Rev.* 2011(10):CD004184.

13. De Giorgi A, Mallozzi Menegatti A, Fabbian F, Portaluppi F, Manfredini R. Circadian rhythms and medical diseases: does it matter when drugs are taken? *Eur J Intern Med.* 2013;24(8):698-706.

14. Smolensky MH, Hermida RC, Ayala DE, Portaluppi F. Bedtime hypertension chronotherapy: concepts and patient outcomes. *Curr Pharm Des.* 2015;21(6):773-790.

15. Roush GC, Fapohunda J, Kostis JB. Evening dosing of antihypertensive therapy to reduce cardiovascular events: a third type of evidence based on a systematic review and meta-analysis of randomized trials. *J Clin Hypertens (Greenwich).* 2014;16(8):561-568.

16. Tatti P, Pahor M, Byington RP, et al. Outcome results of the Fosinopril Versus Amlodipine Cardiovascular Events Randomized Trial (FACET) in patients with hypertension and NIDDM. *Diabetes Care.* 1998;21(4):597-603.

17. Black HR, Elliott WJ, Grandits G, et al. Principal results of the Controlled Onset Verapamil Investigation of Cardiovascular End Points (CONVINCE) trial. *JAMA.* 2003;289(16):2073-2082.

18. Zhang R, Lahens NF, Ballance HI, Hughes ME, Hogenesch JB. A circadian gene expression atlas in mammals: implications for biology and medicine. *Proc Natl Acad Sci U S A.* 2014;111(45):16219-16224.

19. Hermida RC. Sleep-time ambulatory blood pressure as a prognostic marker of vascular and other risks and therapeutic target for prevention by hypertension chronotherapy: Rationale and design of the Hygia Project. *Chronobiol Int.* 2016;33(7):906-936.

20. Rorie DA, Rogers A, Mackenzie IS, et al. Methods of a large prospective, randomised, open-label, blinded end-point study comparing morning versus evening dosing in hypertensive patients: the Treatment In Morning versus Evening (TIME) study. *BMJ Open.* 2016;6(2):e010313.

21. Van Spall HG, Toren A, Kiss A, Fowler RA. Eligibility criteria of randomized controlled trials published in high-impact general medical journals: a systematic sampling review. *JAMA.* 2007;297(11):1233-1240.

22. Hansson L, Hedner T, Dahlof B. Prospective randomized open blinded end-point (PROBE) study. A novel design for intervention trials. Prospective Randomized Open Blinded End-Point. *Blood Press.* 1992;1(2):113-119.

23. Grieshaber MC, Flammer J. Blood flow in glaucoma. *Curr Opin Ophthalmol.* 2005;16(2):79-83.

24. Hayreh SS. Role of nocturnal arterial hypotension in the development of ocular manifestations of systemic arterial hypertension. *Curr Opin Ophthalmol.* 1999;10(6):474-482.

25. Hayreh SS, Zimmerman MB, Podhajsky P, Alward WL. Nocturnal arterial hypotension and its role in optic nerve head and ocular ischemic disorders. *Am J Ophthalmol.* 1994;117(5):603-624.

26. Krasinska B, Karolczak-Kulesza M, Krasinski Z, et al. Effects of the time of antihypertensive drugs administration on the stage of primary open-angle glaucoma in patients with arterial hypertension. *Blood Press.* 2012;21(4):240-248.

27. Quan H, Sundararajan V, Halfon P, et al. Coding algorithms for defining comorbidities in ICD-9-CM and ICD-10 administrative data. *Med Care.* 2005;43(11):1130-1139.

28. Tonelli M, Wiebe N, Fortin M, et al. Methods for identifying 30 chronic conditions: application to administrative data. *BMC Med Inform Decis Mak.* 2015;15:31.

29. Kokotailo RA, Hill MD. Coding of stroke and stroke risk factors using international classification of diseases, revisions 9 and 10. *Stroke; a journal of cerebral circulation.* 2005;36(8):1776-1781.

30. Mueller PS, Montori VM, Bassler D, Koenig BA, Guyatt GH. Ethical issues in stopping randomized trials early because of apparent benefit. *Ann Intern Med.* 2007;146(12):878-881.

Log of BedMed-Frail Amendments

**Date of Submission (D-M-Y)**	**Date of Acceptance (D-M-Y)**	**Summary of Amendment**<br>(Wording as submitted to the ethics board)
05-11-2019	08-11-2019	Our study has not yet started randomization. We wish to adjust our methods now to deal with an anticipated logistic issue related to how we will keep track of which patients are randomized to the intervention and which patients are randomized to control.<br><br>THE LOGISTIC ISSUE:<br>Our study was approved for cluster randomization, with each cluster being a ward or wing of a participating LTC facility (i.e. each facility would have multiple clusters within it). Our current process involves the data steward telling the facility which patients are eligible for the study, and subsequently learning from the facility which ward those patients are on so that the data steward can determine their allocation, record their allocation, and advise the pharmacist of whose medication timing needs to change. This requires dialog between multiple people (data steward, LTC administration, and pharmacist), is not easily automated, has more opportunity for error, and the process is ongoing over the course of the study because of patients being newly admitted (i.e. must take place many times).<br><br>THE SOLUTION:<br>It is much easier for the facility staff and data steward to carry out the study if we switch to patient level randomization / allocation performed by the data steward. In so doing, the data steward automatically and reliably knows the allocation of each patient and needs to send only a single easily automated communication to the pharmacist telling them which patients have been allocated to switch to bedtime BP medications. This would not materially change anything for the residents, who would still have a 50/50 chance of randomization and who would still have exactly the same opportunity to opt out. The data steward will still ask the facility to update the level of care of eligible residents periodically (for use as a covariate in analysis), but this can now be done less frequently and can be automated.<br>ADDITIONAL CHANGE:<br>We have also taken the opportunity of submitting this amendment to update our description of the flow of information between the facility, the research team, and the data steward needed to communicate:<br><br>1) Which patients have opted out of the study (and hence ensure those patients BP medication timing does not change).<br>• All residents and families will be notified of the trial and will have a minimum of 30 days to opt out of the trial by notifying caregivers, administrators, or the study team directly.<br>• Caregivers and administrators will notify the study team, and the study team will in turn notify the data steward, who will remove the resident from the study and ensure their name is not forwarded to the pharmacist for BP medication timing change.

		2) Which residents are currently residing in each facility prior to the initial randomization.<br>• The data steward will be requesting a list of all residents from each facility prior to the initial randomization so that they can verify they are accurately detecting all current residents prior to attempting to identify those who are eligible. The study team does not receive this information.<br><br>3) Identifying the level of care of eligible residents for use in baseline characteristics and as a covariate in analysis.<br>• To accomplish this, the data steward will periodically send via secure e-mail link a spreadsheet of eligible residents to an administrator designated by each facility. That administrator will then enter the level of care designation for each of their residents and return this to the data steward. Each facility designate will only see the names of their own residents.
10-02-2020	11-02-2020	In order to allow for AHS to utilize AHS datasets, we need to add them as a Research location under section 1.6 (Research Locations).
21-03-2022	22-03-2022	Change in study coordinator contact information on documentation.
24-01-2023	30-01-2023	A new process is being added: AHS and Alberta Health administrative data will be utilized between April 2002 and December 2025 for the purpose of determining patient characteristics and outcomes. Specifically, PIN will be used to determine Blood Pressure medication timing in the 30 days before randomization up to 6 months post randomization by using the medication instructions held within PIN using appropriate keyword searches.
25-04-2023	25-04-2023	1) We needed to change from Janis's name to my name (new RC on project).<br>2) We noticed that the text speaks to the whole facility potentially changing timing, whereas participants are randomized at the patient level and only some individuals will have their timing changed. This likely happened because the original study protocol was for a cluster randomized trial. Hence we are asking to change a few words to show that individuals potentially have their timing changed, not the whole facility.<br>3) We also took the opportunity to change any reference to long term care, or supportive living, to "continuing care" which encompasses both levels of care. This will make it easier for some facilities, who have both supportive living and long term care sites, so that they only need to deal with a single version of the notification letter.<br>4) There was also a typo where an apostrophe was missing - and is now added.

08-10-2024	11-10-2024	1. Sub-study: During the course of data analysis, the primary outcome of 'major adverse cardiovascular events' trended to benefit (although not statistically significant) and 'unplanned all-cause hospitalization/emergency department (ED) visit' saw a statistically significant reduction of 28% (p = 0.01).To further explore this finding, long term care facility residents will be observed for an additional 1 year post planned final analysis with a repeat analysis of all outcomes conducted in March of 2025. Several months after the end of data collection we reached out to all participating facilities and were told that none of the participants' antihypertensive medication timings had been changed. We would like to ask them to continue with those allocations, given we have demonstrated no harm and the possibility of benefit for bedtime prescribing which requires further exploration.<br><br>2. Chronotype category analysis: Given a recent antihypertensive timing trial (TIME) demonstrated that chronotype, which is defined by the time of day people are most active (often referred to as early birds or night owls), might influence the effect of antihypertensive timing on cardiovascular events, we would like to explore this with the BedMed-Frail data as well. Although chronotype itself is not available in the RAI-MDS data, there are three things which we feel may create a rough approximation for chronotype. These are the description of the time of day when patients are active (morning, midday, evening), problems with insomnia, and use of sleeping pills. We would define early birds as being those who are active in the morning and not the evening and we would define night owls as being those who are active in the evening but not the morning or those who have insomnia or are using sleeping pills. We would like to repeat the analysis of the primary outcome and the analysis of all cause unplanned hospitalization/ED visits using early bird and night owl as covariates in the survival analysis carried out for each of these outcomes, as well as including an interaction term between allocation and each of these chronotype covariates. In so doing, we would be looking to see if the hazard ratio and p-value for those covariates suggests an influence of chronotype on the effects of medication timing on mortality and morbidity.

TABLE OF CONTENTS

This supplement contains the following items:

1. BedMed-Frail Statistical Analysis Plan (The only version of the statistical analysis plan – created in advance of the interim analysis and submitted for publication on April 17, 2023)
2. Attestation and signatures from the Principal Investigator and Senior Statistician

*Note: The final version of the BedMed-Frail protocol, along with the final statistical analysis plan, was published on Aug 1, 2023 as: *Garrison SR, Youngson E, Perry DA, Campbell FN, Kolber MR, Korownyk C, Allan GM, Green L, Bakal J. Bedtime versus morning use of antihypertensives in frail continuing care residents (BedMed-Frail): protocol for a prospective, randomised, open-label, blinded end-point pragmatic trial. BMJ Open. 2023 Aug 1;13(8):e074777. doi: 10.1136/bmjopen-2023-074777. PMID: 37527898; PMCID: PMC10394547.*

BedMed-Frail Statistical Analysis Plan

Adaptive Randomization (conversion to modified intention-to-treat)

Participating facilities are volunteering to support our trial with no financial compensation, and "fitting it in" with other work. For several facilities, COVID-19, other infectious outbreaks, and implementation of other facility initiatives has led to long delays before the facility pharmacist was able to act on their allocation list. For a few facilities this delayed implementation by several months. Given 1) we are placing an equal emphasis on detecting harm, 2) long implementation delays make both groups look more similar and reduce the ability to detect harm, and 3) no action of any kind was taken related to the facility's allocation list in this interval, we have adapted the trial to consider the date of each facility's initial randomization to be the date the allocation list was actually acted upon, and not the date it was generated. This date was determined by the AHS data analyst, using electronic pharmacy dispensing data, to be the day before the first switch of an eligible patient's BP medications from morning to bedtime. Individuals who, on the date of implementation, were no longer resident in the facility, hospitalized, or without once daily BP meds, were excluded from the trial.

Outcomes

Unless otherwise stated, all outcomes are recorded over the duration of the study.

Primary:

Major adverse cardiovascular events (MACE)

- Defined as first occurrence of either all-cause death or hospital admission / emergency department (ED) visit for acute coronary syndrome / myocardial infarction (MI), stroke, or congestive heart failure (CHF)

Secondary Efficacy:

1. Each component of the primary outcome individually.
2. All-cause unplanned hospitalization / ED visit (per Canadian Institute for Health Information DAD Abstracting Manual's definition of "urgent/emergent" – which excludes elective surgeries or booked procedures / planned follow-up care)[1]

Secondary Safety:

1. Non-vertebral fracture
2. Fall in the last 30 days*
3. Urinary incontinence (≥ "occasionally incontinent" = 2 or more times per week)*
4. Partial or full thickness decubitus skin ulceration (i.e. stages 2 to 4)*

Cognitive / Behavioral

5. "Deteriorated cognition relative to status 90 days prior"*
6. "Behavioral symptoms that are present a minimum of 4 days per week and not easily altered in the last 7 days" (includes wandering, verbal abuse, physical abuse, socially inappropriate or disruptive behavior, and resisting care)*

7. Receipt of antipsychotic medication or physical restraints (chair, trunk, or limb restraints, does not include bedrails) in the last 7 days*
8. Receipt of anti-anxiety medication on ≥3 of the last 7 days*
9. Receipt of a bedtime sleeping pill on ≥3 of the last 7 days*
10. "Indicators of depression or anxiety almost daily" in the last 30 days (applies to 16 discrete mood and anxiety related observations of the resident, any one of which being listed as present 6 or more days per week would qualify)*

* Safety outcomes 2 – 10 will only be examined in LTC residents (and not Level 4 or 4D residents), as these measures are not as regularly collected on those who reside in facilities with a lower level of care. Each are binary outcomes as recorded by usual care nurses in the Quarterly RAI-MDS report in the 3- to 6-month window post-randomization (on average these assessments should take place roughly 135 days post-randomization, and the assessment closest to 135 days is chosen when more than one is available)

Cost:

1. Acute care costs (estimated from each hospital / ED admission's resource intensity weight and length of stay)
2. Total cost of care (acute care costs + medication costs + physician billings + nursing / facility costs; with nursing / facility costs being estimated from level of care and duration of stay).

Process:

1. Proportion of BP medication doses taken at bedtime post-randomization, broken down on a monthly basis (with medications dosed more than once a day being considered as ½ dose in the AM and ½ dose in the PM for this calculation).
2. Use of at least one once-daily BP medication at Bedtime.
   - Note: Both of these process outcomes are determined, for each facility and for the study as a whole, using the "directions for use" field in Pharmaceutical Information Network (PIN) data. Each will be presented as a monthly time series, and will use only those randomized participants who are alive and resident at that facility at the end of each respective month.

Statistical Analysis

Intension-to-treat assumptions

Loss to follow-up: If participants change continuing care facilities within Alberta, we will no longer be able to control their medication timing, which is likely to default to morning use upon entering a new facility. As such, we will censor residents who change facilities on the first day of residence in the new continuing care facility. However, we will not censor patients transferred to "inpatient LTC" at a local hospital, as that is likely to represent an escalation in care due to acute illness, or diminished capacity. If such individuals improve, they may return to the original continuing care facility and we can continue to follow them. If not, the transfer to "inpatient LTC" likely signals a

deterioration of health that may precede adverse health outcomes that would be important to capture. If residents change their level of care, but remain in the same facility (i.e. move to a different floor, or wing, which has a different level of care), we will continue to follow them, as their medication timing is unlikely to change.

Withdrawal: Residents who do not want to participate in BedMed-Frail are generally “opted out” (i.e. excluded) prior to randomization. Should we be approached, by a resident, their family, or their providers, to opt-out a resident after randomization, that is likely to be prompted by an attempted timing change, and hence a more likely occurrence for those allocated to bedtime. To avoid the introduction of attrition bias, all post-randomization opt-outs will be included and analyzed per their allocation unless we are explicitly asked not to follow their electronic health data.

Missing MDS data: For each analysis, we will use the post-randomization MDS nursing report closest to day 135 (tie going to the later date). If there are no such post-randomization reports we will use the report immediately preceding randomization. If no reports of any kind are available (which should not occur as a baseline MDS report is a routine part of every admission), we will exclude the participant from the analysis.

Nonadherence: Nonadherence to allocation will not exclude participants from any of our planned analyses. However, as outlined in Sensitivity Analysis below, we will conduct a per-protocol analysis to determine whether results differ when adherence to allocation is highest.

Baseline Characteristics

Comorbidities are determined using all available data in the linked healthcare databases (from 2002 until date of randomization), and require either one hospital diagnosis or two community diagnoses. Chronic kidney disease (CKD) is additionally defined by laboratory data when eGFR < 40 $mL/min/1.73m^2$ on 2 occasions > 3 months apart.

Selecting regression covariates

Analyses of dichotomous outcomes will use a maximum of 1 covariate per 10 outcomes, and analyses of continuous outcomes will use a maximum of 1 covariate per 20 randomized subjects. The covariate list for each analysis is predefined in table 2, and all are measured at baseline. We will always use the maximum number of covariates possible, selected in the order given (i.e. we will not undertake stepwise addition or subtraction). As we go down the covariate list, if one covariate has too many levels to be accommodated by the remaining number of outcomes or participants (e.g. facility, or level of care) we will skip it and go to the next covariate in the list.

Table 2 Analysis Plan

**Outcome**	**Method**	**Baseline Covariates**
Primary		
Major adverse cardiovascular events	Cox Proportional Hazards	Age <70, Gender, Facility/Level of Care specific mortality rate (prior 3 years), New admission*,

		Old admission†, Days hospitalized in prior 6 months, Number of ED visits in prior 6 months, Charlson score, Facility, Total number of non-BP medications, Total number of BP medications, CHF, COPD, CKD, stroke, diabetes, CAD, sleep apnea
Secondary Efficacy		
All-cause mortality	Cox Proportional Hazards	Same as for primary outcome
Hospitalization for stroke<br><br>Hospitalization for MI/ACS<br><br>Hospitalization for CHF	Cox Proportional Hazards	Facility/Level of Care specific hospitalization rate (prior 3 years)
All-cause unplanned hospitalization / ED visit	Cox Proportional Hazards	Facility/Level of Care specific hospitalization rate (prior 3 years), New admission*, Old admission)†, Days hospitalized in prior 6 months, Number of ED visits in prior 6 months, Charlson score, Age < 70, Gender, Total number of non-BP medications, Total number of BP medications, CHF, COPD, CKD, Diabetes
Secondary Safety (non-cognitive)		
Non-vertebral fracture	Cox Proportional Hazards	Facility/Level of Care specific non-vertebral fracture rate (prior 3 years), prior non-vertebral fracture, Gender, Age < 70, New admission*, Old admission†
Fall in the last 30 days	Poisson Regression	Facility/Level of Care specific prior % fallers‡, Facility/Level of Care specific non-vertebral fracture rate (prior 3 years), New admission*, Old admission†, Gender
Urinary incontinence	Poisson Regression	Facility/Level of Care specific prior % incontinent‡, New admission*, Old admission†, Age < 70, stroke, gender, diabetes
Full thickness skin ulceration (stage 3 or 4)	Poisson Regression	Facility/Level of Care specific prior % ulcerated,‡ New admission*, Old admission†, Stroke, Number of BP medications, Age < 70, gender
Secondary Safety (cognitive-behavioral)		

"Deteriorated cognition relative to status 90 days prior"	Poisson Regression	Facility/Level of Care specific prior % deteriorated‡, Age <70, New admission*, Old admission†, gender
"Behavioral symptoms that are present a minimum of 4 days per week and not easily altered" (Includes wandering, verbal abuse, physical abuse, socially inappropriate or disruptive behavior, and resisting care)	Poisson Regression	Facility/Level of Care specific prior % behavior problems‡, New admission*, Old admission†, Gender, Age < 70, Days hospitalized in prior 6 months, Number of ED visits in prior 6 months, Total number of non-BP medications, Total number of BP medications, Charlson score
Receipt of antipsychotic medication or physical restraints in the last 7 days	Poisson Regression	Same as above
Receipt of anti-anxiety medication on ≥3 of the last 7 days	Poisson Regression	Same as above
Receipt of a bedtime sleeping pill on ≥3 of the last 7 days	Poisson Regression	Same as above
"Indicators of depression or anxiety almost daily" in the last 30 days	Poisson Regression	Same as above
Cost		
Acute care costs<br><br>Total cost of care	Multiple Linear Regression	Facility, Level of Care§, Age <70, New admission*, Old admission†, Gender, Days hospitalized in prior 6 months, Number of ED visits in prior 6 months, Charlson score, Total number of non-BP medications, Total number of BP medications, CHF, COPD, diabetes, CAD, stroke, CKD

* Resident in current facility less than 60-days at time of randomization
† Resident in current facility more than 3-years at time of randomization
‡ % of all RAI-MDS reports from this facility over the last 3-years indicating this outcome
§ In order of increasing care, this includes: Levels 3, 4, 4D, and 5 (as described in the manuscript under Setting and Recruitment)

Subgroup Analyses

We will repeat the primary outcome analysis for those with and without the following baseline characteristics: Male sex, resistant hypertension (defined as ≥ 3 antihypertensive medications), CHF, CKD, diabetes and (separately) use of the major BP medication classes (ACEI, ARB, CCB, diuretic, beta-blocker).

Sensitivity Analyses

1) We will conduct a per-protocol analysis by excluding residents from any facility where fewer than 60% of medication doses are taken at bedtime in the intervention group 6-months post implementation.
2) We will conduct a full intention-to-treat analysis by including everyone who was initially assigned an allocation, even if they became ineligible (e.g., died) while waiting for their facility to act on the allocation list.

REFERENCES

1. Canadian Institute of Health Information. DAD/NACRS Abstracting Manual (web tool) https://www.cihi.ca/en/dadnacrs-abstracting-manual-web-tool Accessed Feb 2 2023

6-60 University Terrace
Edmonton, Alberta, Canada T6G 2T4
Tel: 780.248.1853
Fax: 780.248.2039
scott.garrison@ualberta.ca

Sept 20, 2024

We the undersigned confirm that all analyses carried out for the BedMed-Frail randomized controlled trial make use of all available subjects and adhere completely to the detailed statistical analysis plan published as part of the BedMed-Frail protocol on Aug 1, 2023 and cited as:

Garrison SR, Youngson E, Perry DA, et al. Bedtime versus morning use of antihypertensives in frail continuing care residents (BedMed-Frail): protocol for a prospective, randomised, open-label, blinded end-point pragmatic trial. BMJ Open 2023;13(8):e074777. DOI: 10.1136/bmjopen-2023-074777.

J Bakal

Jeffrey A Bakal, PhD, Pstat
BedMed-Frail Co-Investigator and Senior Statistician

Sept 20, 2024
Date

Scott Garrison

Scott R Garrison MD, PhD
BedMed-Frail Principal Investigator

Sept 20, 2024
Date
