# Supplementary material for: Bedtime vs Morning Antihypertensive Medications in Frail Older Adults: The BedMed-Frail Randomized Clinical Trial
Source: JAMA Netw Open. 2025 May 12;8(5):e2513812. doi: 10.1001/jamanetworkopen.2025.13812 (PMC12070236; doi:10.1001/jamanetworkopen.2025.13812)
Supplement: Supplement 2. — eAppendix 1. BedMed-Frail Committees, Investigators, and Staff eAppendix 2. Definitions, Appropriateness of the Statistical Analyses, and Changes to Secondary Outcomes eAppendix 3. Electronic Health Data (ICD-9/ICD-10) Outcome Definitions eTable 1. Adapted Randomization Dates eTable 2. Continuing Care Ward Characteristics eTable 3. Percentage of Antihypertensive Medication Doses at Bedtime for Individual Medications by Class of Antihypertensive (Baseline and 6-Months) eFigure 1. Mean % of Antihypertensive “Doses” in the Evening (Primary Analysis Participants) eFigure 2. Mean % of Antihypertensive Doses in the Evening (Per-Protocol Participants) eTable 4. Baseline Patient Characteristics Comparing Residents Included vs Excluded From Per-Protocol Analysis (Based on Exclusion of Facilities With < 60% Adherence) eTable 5. Primary and Secondary Outcomes—Per-Protocol Analysis eTable 6. Baseline Patient Characteristics Comparing Residents Included vs Excluded From Full Intention-to-Treat Analysis eTable 7. Primary and Secondary Outcomes—Full Intention-to-Treat Analysis eTable 8. Analysis of Unplanned All-cause Hospitalization/ED Visits as a Continuous Variable (Poisson Regression), Counting All Events During Follow-Up Period eTable 9. Unadjusted Hazard Ratios for Primary and Secondary Outcomes eFigure 3. Effect of Medication Timing on All-Cause Death [file jamanetwopen-e2513812-s002.pdf]

Supplemental Online Content

Garrison SR, Youngson ERE, Perry DA, et al. Bedtime vs morning antihypertensive medications in frail older adults: the BedMed-Frail randomized clinical trial. *JAMA Netw. Open*. 2025;8(5):e2513812. doi:10.1001/jamanetworkopen.2025.13812

eAppendix 1. BedMed-Frail Committees, Investigators, and Staff
eAppendix 2. Definitions, Appropriateness of the Statistical Analyses, and Changes to Secondary Outcomes
eAppendix 3. Electronic Health Data (ICD-9/ICD-10) Outcome Definitions
eTable 1. Adapted Randomization Dates
eTable 2. Continuing Care Ward Characteristics
eTable 3. Percentage of Antihypertensive Medication Doses at Bedtime for Individual Medications by Class of Antihypertensive (Baseline and 6-Months)
eFigure 1. Mean % of Antihypertensive “Doses” in the Evening (Primary Analysis Participants)
eFigure 2. Mean % of Antihypertensive Doses in the Evening (Per-Protocol Participants)
eTable 4. Baseline Patient Characteristics Comparing Residents Included vs Excluded From Per-Protocol Analysis (Based on Exclusion of Facilities With < 60% Adherence)
eTable 5. Primary and Secondary Outcomes—Per-Protocol Analysis
eTable 6. Baseline Patient Characteristics Comparing Residents Included vs Excluded From Full Intention-to-Treat Analysis
eTable 7. Primary and Secondary Outcomes—Full Intention-to-Treat Analysis
eTable 8. Analysis of Unplanned All-cause Hospitalization/ED Visits as a Continuous Variable (Poisson Regression), Counting All Events During Follow-Up Period
eTable 9. Unadjusted Hazard Ratios for Primary and Secondary Outcomes
eFigure 3. Effect of Medication Timing on All-Cause Death

This supplemental material has been provided by the authors to give readers additional information about their work.

© 2025 Garrison SR et al. *JAMA Network Open*.

eAppendix 1: BedMed-Frail Committees, Investigators, and Staff

Steering Team: Scott Garrison (Overall Principal Investigator), Erik Youngson, Danielle Perry, Jeffrey Bakal

ICD9/10 Coding Review Committee: Jessica Kirkwood, Roni Kraut, Scott Garrison

Data and Safety Monitoring Board: Jim Wright (Chair), Tom Perry, Marco Perez, Benji Heran, Mohamed Ben-Eltirki

Study Statistician/Programmer: Erik Youngson

Research Coordinators: Farah Campbell, Janis Cole, Nicole Olivier

Participating Facilities:

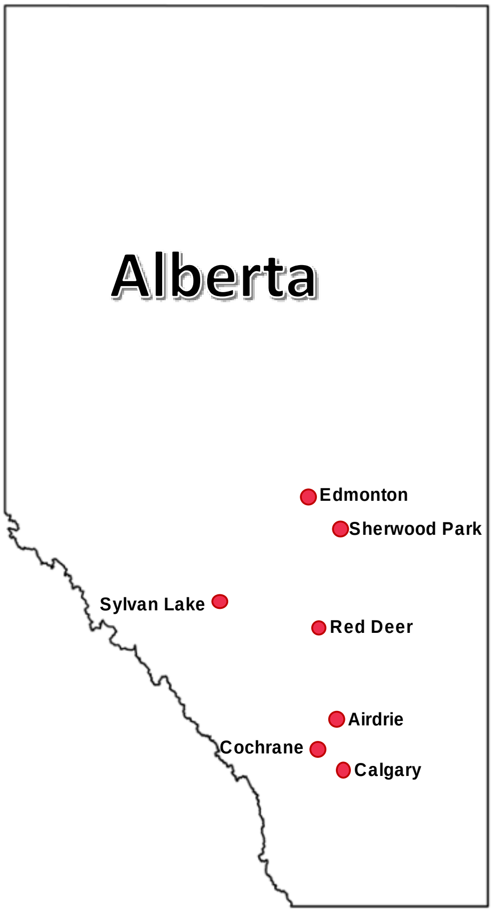

EDMONTON
- Facility A – LTC *(99)*
- Facility B – LTC *(137)*
- Facility C – LTC *(69)*
- Facility D – LTC *(147)*
- Facility E – LTC *(53)*

SHERWOOD PARK
- Facility F – LTC *(100)*

SYLVAN LAKE
- Facility G – DSL4 *(21)*
- Facility H – LTC *(40)*

RED DEER
- Facility I – LTC *(112)*

AIRDRIE
- Facility J – LTC *(74)*

COCHRANE
- Facility K – LTC *(78)*

CALGARY
- Facility L – LTC *(416)*
- Facility M – LTC *(60)*
- Facility N – LTC *(145)*
- Facility O – LTC *(80)*
- Facility P – DSL4 *(59)*
- Facility Q – *DSL4D (36)*

*Numbers in brackets are the number of facility beds, not the number of participants enrolled. LTC = Long Term Care, DSL4 = “Designated Supportive Living 4”, DSL4-D = “Designated Supportive Living 4-Dementia”. Care level descriptions are provided in the published protocol.

© 2025 Garrison SR et al. *JAMA Network Open.*

eAppendix 2: Definitions, Appropriateness of the Statistical Analyses, and Changes to Secondary Outcomes

Lost to follow-up & withdrawal: Although we had full capture of electronic health data throughout the province of Alberta, we were not able to control medication timing once residents left the facility in which they were randomized. Hence, we censored all residents leaving their facility of randomization on the date of discharge. This was the sole source of what would conventionally be termed withdrawal or lost to follow-up.

Proportional hazards assumptions: For each comparison of the primary and secondary outcomes, the plausibility of the proportional hazards assumption was assessed by including a time-treatment interaction term in the Cox model (time log transformed). Given the interaction p –values were not statistically significant, the proportional hazards assumption was satisfied.

Changes to Secondary Outcomes: Some changes, and additions, were made to secondary outcomes *while fully blinded to all trial outcomes*.

1. **Anti-anxiety medication use, and sleeping pill use, were changed from continuous variables** (counting the number of days used in the last week), **to binary variables** (used 3 or more days in the last week). RATIONALE: This change was made to produce variables that were easier to analyze and interpret.
2. **Total cost of care was added as an additional outcome.** RATIONALE: This new outcome was added to augment the cost analysis, which previously only looked at acute care costs.
3. **The definition of skin ulceration was changed from full thickness (stage 3-4) to partial or full-thickness (stage 2-4).** RATIONALE: As the total number of full thickness ulcers was small, this change was made to increase the number of such events and improve the ability to detect a difference between groups, should one exist.
4. **We retained the composite outcome "use of antipsychotic medication or physical restraints" but removed each component on its own.** RATIONALE: Numbers of events were small for both and unlikely to be significantly different on their own.
5. **We changed our 6-month process outcomes** from an assessment of 1) percentage of once-daily BP medication taken per allocation, 2) number of BP meds, and 3) number of BP meds used more than once a day to *proportion of BP medication doses taken at bedtime* and *use of at least one once-daily medication at bedtime.* RATIONALE: We made this change once we gained access to the directions for use field of the pharmacy dispensing data as we could now more easily track medication timing – both at 6-months and on a monthly basis up to 1-year post randomization. The new process outcomes also lent themselves to graphical representation, and incorporated BP medications used twice daily.

© 2025 Garrison SR et al. *JAMA Network Open.*

eAppendix 3: Electronic Health Data (*ICD-9/ICD-10*) Outcome Definitions

Overview: The following ICD9/10 diagnostic code definitions were applied to the Alberta linked health databases to identify trial outcomes. While individual participant level data was not adjudicated (i.e. all identified outcomes were accepted), the resulting range of ICD9/10 codes identifying trial outcomes was reviewed by a panel of 3 physicians to ensure no coding errors.

Events	ICD9/10 Definitions Used
**Myocardial infarction/acute coronary syndrome**<br>Hospitalization or ED visit	**ICD10:** I20%, I21%, I22%, I24%<br>**ICD9:** 410%, 411%, 412%, 413%, 414%
	**Note:** Only the primary diagnosis qualified
**Stroke**<br>Hospitalization or ED visit	**ICD10:** H34.1%,<br>I60%, I61%, I63%, I64%<br>**ICD9:** 430%, 431%, 432%, 433%, 434%,<br>362.3%
	**Note:** Only the primary diagnosis qualified
**Congestive Heart Failure**<br>Hospitalization or ED visit	**ICD10:** I50%<br>**ICD9:** 428%
	**Note:** Only the primary diagnosis qualified
**Non-Vertebral Fracture**	**ICD10:**<br>S02%<br>S22.2% - S22.9%<br>S32.1% - S32.6%<br>S42%, S52%, S62%<br>S72.0%, S72.1%, S72.2%<br>S72.3% - S72.9%<br>S82%, S92%, T02%, T10%, T12%<br>T14.2%<br><br>**ICD9:** N/A
	**Event qualified by:**<br>**Note:** ANY diagnosis qualified, not just the primary

© 2025 Garrison SR et al. *JAMA Network Open*.

eTable 1: Adapted Randomization Dates

Continuing Care Ward*	Number Randomized	Allocated to Bedtime	Initial Randomization Date	Revised Randomization Date	Difference in days
Facility 1	21	13	13-May-20	25-May-20	12
Facility 2	6	4	15-Jul-20	27-Jul-20	12
Facility 3	29	11	14-Dec-20	16-Dec-20	2
Facility 4	27	11	14-Dec-20	20-Dec-20	6
Facility 5	17	12	14-Dec-20	21-Mar-21	97
Facility 6 & 7	35	18	14-Dec-20	11-Apr-21	118
Facility 8	15	6	17-May-21	29-Aug-21	104
Facility 9	9	5	17-May-21	12-Sep-21	118
Facility 10	20	12	15-Dec-21	29-Dec-21	14
Facility 11	22	11	15-Feb-22	16-Feb-22	1
Facility 12	24	12	26-Oct-22	27-Oct-22	1
Facility 13	28	19	17-Oct-22	09-Nov-22	23
Facility 14	122	67	15-Sep-22	10-Jan-23	117
Facility 15	33	14	15-Sep-22	25-Jan-23	132
Facility 16	50	26	14-Apr-23	16-May-23	32
Facility 17	27	11	22-Jun-23	29-Jun-23	7

* To ensure de-identification of participating facilities, individual facilities are intentionally listed as Facility A, Facility B …Facility Q in Section 1 of the supplemental appendix, and Facility 1, Facility 2 …Facility 17 in this table. There is no correspondence in the order in which facilities are listed – i.e. Facility A and Facility 1 are not the same entities.

© 2025 Garrison SR et al. *JAMA Network Open*.

eTable 2: Continuing Care Ward Characteristics

**Characteristic**	**Overall**
Number of distinct continuing care wards	**N = 17**
Level of Care* — no. (%)	
DSL4	2 (11.8)
DSL4-D	1 (5.9)
LTC	14 (82.4)
Total number of beds, median (IQR)	78 (59, 112)
Outcome rates, events per 100 patient-yrs† — median (IQR)	
Mortality	32.0 (23.3, 40.7)
Hospitalization/ED visits	36.2 (25.5, 49.9)
Non-vertebral fracture	4.5 (3.2, 5.3)
Outcome prevalence‡, percentage — median (IQR)	
Fall in past 30 days	17.6 (14.5, 20.7)
Stage 3-4 skin ulcer	3.0 (2.1, 4.1)
Urinary incontinence	88.3 (81.1, 90.2)
Deteriorated cognition in last 90 days	7.9 (6.1, 13.1)
Antipsychotic medication or physical restraints used	22.4 (18.7, 26.6)
Anti-anxiety medications used on ≥3 of the last 7 days	5.4 (2.7, 8.3)
Bedtime sleeping pill used on ≥3 of the last 7 days	9.3 (6.3, 14.2)
Indicators of depression/anxiety almost daily	10.5 (5.7, 21.6)
Problem behaviours	14.0 (8.8, 19.6)

*Levels of care are described in detail in the published protocol. LTC = Long-Term Care, which are facilities with the highest level of support. DSL4 = Designated Supportive Living Level 4, where residents are somewhat more independent. DSL4-D = DSL4 but with extra precautions to deal with dementia (e.g. precautions to prevent wandering).

†Calculated using all residents on the same ward in the 3-years prior to that facilities randomization date.

‡Represents the percentage of all RAI-MDS reports where the outcome is positive in the 3-years prior to that facility's randomization date.

© 2025 Garrison SR et al. *JAMA Network Open.*

eTable 3: Percentage of Antihypertensive Medication Doses at Bedtime for Individual Medications by Class of Antihypertensive (Baseline and 6-Months)

Medication Class	Treatment (Bedtime)		Control (Morning)	
	% of doses at bedtime	N included	% of doses at bedtime	N included
**All medication classes**				
Baseline	13.1	394	15.9	382
6-months	66.9	309	15.6	300
**Angiotensin converting enzyme inhibitors**				
Baseline	17.1	145	16.9	148
6-months	73.9	113	16.2	114
**Angiotensin receptor blockers**				
Baseline	9.5	116	10.4	101
6-months	65.2	99	10.7	84
**Diuretics**				
Baseline	1.4	73	4.8	63
6-months	27.8	54	3.1	48
**Calcium channel blockers**				
Baseline	20.4	169	24.3	180
6-months	72.7	140	23.8	146
**Beta-blockers**				
Baseline	8.6	87	15.2	69
6-months	71.0	62	14.8	54

© 2025 Garrison SR et al. *JAMA Network Open*.

eFigure 1. Mean % of antihypertensive “doses” in the evening (primary analysis participants)

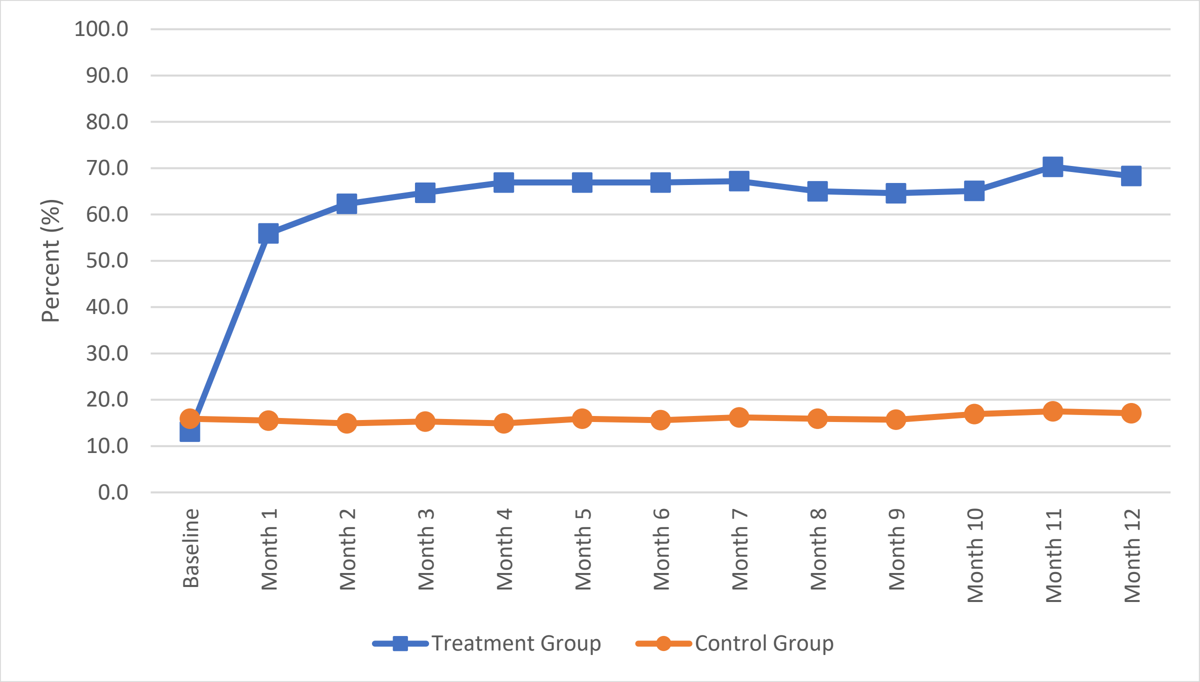

Figure shows the mean percentage of all antihypertensive medications ingested in the evening (Bedtime or dinner). For this calculation medications taken twice daily, or more frequently, were considered to be taken as ½ of their antihypertensive “dose” in the morning, and another ½ in the evening. Hence a participant taking ramipril in the morning and metoprolol twice daily would have (0 + 0.5)/2 = 25% of antihypertensive “doses” in the evening.

© 2025 Garrison SR et al. *JAMA Network Open*.

eFigure 2: Mean % of antihypertensive doses in the evening (per-protocol participants)

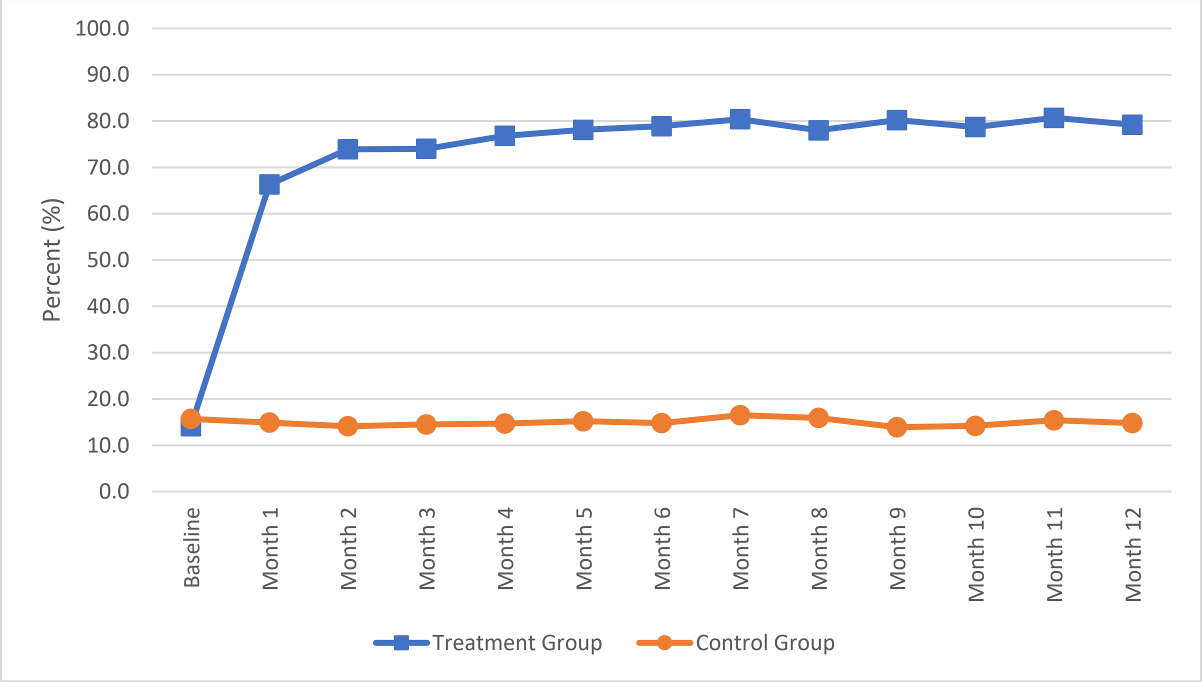

Figure shows the mean percentage of all antihypertensive medications ingested in the evening (bedtime or dinner). For this calculation medications taken twice daily, or more frequently, were considered to be taken as ½ of their antihypertensive "dose" in the morning, and another ½ in the evening. Hence a participant taking ramipril in the morning and metoprolol twice daily would have (0 + 0.5)/2 = 25% of antihypertensive "doses" in the evening.

© 2025 Garrison SR et al. *JAMA Network Open*.

eTable 4: Baseline patient characteristics comparing residents included vs excluded from per-protocol analysis (based on exclusion of facilities with < 60% adherence)

Characteristic	Included in Per-Protocol Analysis N = 559	Excluded from Per-Protocol Analysis N = 217	P Value
Study Group			0.65
Bedtime	281 (50.3)	113 (52.1)	
Morning	278 (49.7)	104 (47.9)	
Age, median (IQR)	87 (79, 91)	90 (84, 94)	<0.001
Age less than 70 years – no. (%)	56 (10.0)	7 (3.2)	0.002
Male – no. (%)	173 (30.9)	41 (18.9)	<0.001
Level of care* – no. (%)			<0.001
DSL4	17 (3.0)	44 (20.3)	
DSL4-D	30 (5.4)	0 (0.0)	
LTC	512 (91.6)	173 (79.7)	
Length of stay in facility, days, median (IQR)	361 (68, 980)	540 (61, 1133)	0.16
Length of stay in facility – no. (%)			
<60 days (new admission)	113 (20.2)	52 (24.0)	
60 days to 3 years	321 (57.4)	108 (49.8)	
>3 years (old admission)	125 (22.4)	57 (26.3)	
Number of BP medications† – no. (%)			0.03
1	344 (61.5)	122 (56.2)	
2	176 (31.5)	72 (33.2)	
3	37 (6.6)	18 (8.3)	
4	1 (0.2)	5 (2.3)	
5	1 (0.2)	0 (0.0)	
Type of BP medications – no. (%)			
Calcium channel blocker (CCB)	239 (42.8)	110 (50.7)	0.05
Angiotensin-converting enzyme (ACE) inhibitor	236 (42.2)	57 (26.3)	<0.001
Angiotensin receptor blockers (ARB)	125 (22.4)	92 (42.4)	<0.001
Beta blocker (BB)	123 (22.0)	33 (15.2)	0.03
Diuretic	89 (15.9)	47 (21.7)	0.06
Other types	5 (0.9)	1 (0.5)	0.54
Number of non-BP medications – no. (%)	6 (4, 8)	6 (4, 8)	0.20
Any days in hospital, prior 6 months – no. (%)	160 (28.6)	52 (24.0)	0.19
Total days in hospital, prior 6 months – mean (SD)	14.7 (30.6)	10.5 (24.2)	0.07
Any ED visit, prior 6 months – no. (%)	162 (29.0)	61 (28.1)	0.81
Number of ED visits, prior 6 months – mean (SD)	0.4 (0.9)	0.4 (0.6)	0.16
Charlson Score, median (IQR)	5 (3, 7)	4 (3, 6)	0.003
Charlson Score, n(%)			0.01
0-1	45 (8.1)	27 (12.4)	

© 2025 Garrison SR et al. *JAMA Network Open*.

2-3	138 (24.7)	59 (27.2)	
4-5	155 (27.7)	71 (32.7)	
6+	221 (39.5)	60 (27.6)	
Comorbidities			
Dementia	501 (89.6)	163 (75.1)	<0.001
Chronic Kidney Disease	263 (47.0)	114 (52.5)	0.17
Diabetes	257 (46.0)	110 (50.7)	0.24
Coronary artery disease	240 (42.9)	67 (30.9)	0.002
Heart failure	185 (33.1)	72 (33.2)	0.98
Stroke	169 (30.2)	55 (25.3)	0.18
Sleep Apnea	146 (26.1)	44 (20.3)	0.09
Chronic obstructive pulmonary disease	139 (24.9)	30 (13.8)	<0.001

*Levels of care are described in detail in the published protocol. LTC = Long-Term Care, which are facilities with the highest level of support. DSL4 = Designated Supportive Living Level 4, where residents are somewhat more independent. DSL4-D = DSL4 but with extra precautions to deal with dementia (e.g. precautions to prevent wandering).
†Combination pills are recorded in the categories of both constituent medications.

© 2025 Garrison SR et al. *JAMA Network Open*.

eTable 5: Primary and Secondary Outcomes—Per-Protocol Analysis

Outcome	Treatment (Bedtime) N = 281		Control (Morning) N = 278		HR/RR (95% CI)
	n (%)	Rate / 100 patient-yr	n (%)	Rate / 100 patient-yr	
**Primary**					
Major adverse cardiovascular event	111 (39.5)	31.6	110 (39.6)	33.2	0.88 (0.67 - 1.15)
**Secondary – Efficacy**					
Primary Outcome Components					
All-cause mortality	109 (38.8)	31.0	108 (38.8)	32.2	0.88 (0.67 - 1.16)
Hospitalization for stroke	3 (1.1)	0.9	4 (1.4)	1.2	0.72 (0.16 - 3.26)
Hospitalization for MI/ACS	0 (0.0)	0.0	2 (0.7)	0.6	-
Hospitalization for HF	4 (1.4)	1.1	5 (1.8)	1.5	0.77 (0.21 - 2.86)
All-cause unplanned hospitalization/ED visit	69 (24.6)	22.7	87 (31.3)	30.7	0.72 (0.52 - 0.996)
**Secondary – Safety***					
Falls/Fractures					
Non-vertebral fracture	6 (2.1)	1.7	6 (2.2)	1.8	0.96 (0.31 – 2.95)
Fall in the past 30 days	44 (17.3)	-	38 (14.8)	-	1.19 (0.77 - 1.85)
Cognitive/Behavioural					
Deteriorated cognition†	28 (11.0)	-	28 (10.9)	-	1.02 (0.61 - 1.73)
Problem behaviours‡	39 (15.3)	-	33 (12.8)	-	1.14 (0.72 - 1.82)
Use of antipsychotic medication or physical restraints§	53 (20.8)	-	71 (27.6)	-	0.73 (0.51 - 1.05)
Indicators of depression or anxiety almost daily‖	55 (21.6)	-	47 (18.3)	-	1.14 (0.77 - 1.69)
Use of anti-anxiety medication¶	23 (9.0)	-	17 (6.6)	-	1.35 (0.72 - 2.53)
Use of bedtime sleeping pill¶	25 (9.8)	-	22 (8.6)	-	1.13 (0.63 - 2.00)
Other					
Partial or Full thickness skin ulceration (stage 2-4)	24 (9.4)	-	31 (12.1)	-	0.76 (0.45 - 1.31)
Urinary incontinence**	227 (89.0)	-	219 (85.2)	-	1.03 (0.86 - 1.24)

*Except for non-vertebral fracture, all safety outcomes are as recorded by nurses on the RAI-MDS 2.0 assessment closest to day 135 post-randomization.
† 'Deteriorated cognition relative to status 90 days earlier' (qualitative global assessment by nurse providing direct care).
‡ 'Behavioural symptoms that are present a minimum of 4 days per week and not easily altered in the last 7 days' (includes wandering, verbal abuse, physical abuse, socially inappropriate or disruptive behaviour and resisting care).
§In the last 7 days. Includes chair, trunk or limb restraints, but does not include bedrails.
‖In the last 30 days. Applies to 16 discrete mood and anxiety related observations of the resident, any one of which being listed as present 6 or more days per week would qualify.
¶On ≥3 of the last 7 days.
** ≥2 times per week.

© 2025 Garrison SR et al. *JAMA Network Open.*

eTable 6: Baseline patient characteristics comparing residents included vs excluded from full intention-to-treat analysis

Characteristic	Included in Intention-to-Treat Analysis N = 776	Excluded from Intention-to-Treat Analysis N = 53	P Value
Study Group			0.41
Bedtime	394 (50.8)	30 (56.6)	
Morning	382 (49.2)	23 (43.4)	
Age, median (IQR)	88 (81, 92)	88 (84, 94)	0.42
Age less than 70 years – no. (%)	63 (8.1)	4 (7.5)	0.88
Male – no. (%)	214 (27.6)	15 (28.3)	0.91
Level of care* – no. (%)			0.76
DSL4	61 (7.9)	4 (7.5)	
DSL4-D	30 (3.9)	1 (1.9)	
LTC	685 (88.3)	48 (90.6)	
Length of stay in facility, days, median (IQR)	350 (64, 984)	498 (175, 1479)	0.02
Length of stay in facility – no. (%)			0.02
<60 days (new admission)	174 (22.4)	4 (7.5)	
60 days to 3 years	429 (55.3)	31 (58.5)	
>3 years (old admission)	173 (22.3)	18 (34.0)	
Number of BP medications† – no. (%)			0.61
1	465 (59.9)	37 (69.8)	
2	249 (32.1)	12 (22.6)	
3	55 (7.1)	4 (7.5)	
4	6 (0.8)	0 (0.0)	
5	1 (0.1)	0 (0.0)	
Type of BP medications – no. (%)			
Calcium channel blocker (CCB)	349 (45.0)	17 (32.1)	0.07
Angiotensin-converting enzyme (ACE) inhibitor	293 (37.8)	19 (35.8)	0.78
Angiotensin receptor blockers (ARB)	217 (28.0)	18 (34.0)	0.35
Beta blocker (BB)	158 (20.4)	11 (20.8)	0.95
Diuretic	135 (17.4)	8 (15.1)	0.67
Other types	6 (0.8)	1 (1.9)	0.39
Number of non-BP medications – no. (%)	6 (4, 8)	6 (4, 8)	0.51
Any days in hospital, prior 6 months – no. (%)	228 (29.4)	17 (32.1)	0.68
Total days in hospital, prior 6 months – mean (SD)	14.7 (30.1)	11.0 (25.0)	0.37
Any ED visit, prior 6 months – no. (%)	233 (30.0)	21 (39.6)	0.14
Number of ED visits, prior 6 months – mean (SD)	0.4 (0.8)	0.6 (0.9)	0.15
Charlson Score, median (IQR)	5 (3, 7)	4 (3, 7)	0.71

© 2025 Garrison SR et al. *JAMA Network Open*.

Charlson Score, n(%)			0.54
0-1	72 (9.3)	3 (5.7)	
2-3	200 (25.8)	16 (30.2)	
4-5	224 (28.9)	12 (22.6)	
6+	280 (36.1)	22 (41.5)	
Comorbidities			
Dementia	663 (85.4)	46 (86.8)	0.79
Chronic Kidney Disease	377 (48.6)	26 (49.1)	0.95
Diabetes	366 (47.2)	20 (37.7)	0.18
Coronary artery disease	307 (39.6)	26 (49.1)	0.17
Heart failure	256 (33.0)	27 (50.9)	0.008
Stroke	223 (28.7)	10 (18.9)	0.12
Sleep Apnea	190 (24.5)	10 (18.9)	0.36
Chronic obstructive pulmonary disease	168 (21.6)	20 (37.7)	0.007

*Levels of care are described in detail in the published protocol. LTC = Long-Term Care, which are facilities with the highest level of support. DSL4 = Designated Supportive Living Level 4, where residents are somewhat more independent. DSL4-D = DSL4 but with extra precautions to deal with dementia (e.g. precautions to prevent wandering).

†Combination pills are recorded in the categories of both constituent medications.

© 2025 Garrison SR et al. *JAMA Network Open*.

eTable 7: Primary and Secondary Outcomes—Full Intention-to-Treat Analysis

Outcome	Treatment (Bedtime) N = 424		Control (Morning) N = 405		HR/RR (95% CI)
	n (%)	Rate / 100 patient-yr	n (%)	Rate / 100 patient-yr	
**Primary**					
Major adverse cardiovascular event	178 (42.0)	29.9	175 (43.2)	31.4	0.91 (0.74 - 1.13)
**Secondary – Efficacy**					
Primary Outcome Components					
All-cause mortality	175 (41.3)	29.2	172 (42.5)	30.6	0.91 (0.73 - 1.13)
Hospitalization for stroke	3 (0.7)	0.5	7 (1.7)	1.2	0.40 (0.10 - 1.57)
Hospitalization for MI/ACS	2 (0.5)	0.3	2 (0.5)	0.4	0.92 (0.13 - 6.42)
Hospitalization for HF	8 (1.9)	1.3	6 (1.5)	1.1	1.24 (0.43 - 3.59)
All-cause unplanned hospitalization/ED visit	120 (28.3)	23.3	140 (34.6)	30.0	0.76 (0.59 - 0.97)
**Secondary – Safety***					
Falls/Fractures					
Non-vertebral fracture	10 (2.4)	1.7	11 (2.7)	2.0	0.86 (0.37 - 2.02)
Fall in the past 30 days	57 (15.3)	-	61 (16.9)	-	0.93 (0.65 - 1.34)
Cognitive/Behavioural					
Deteriorated cognition†	38 (10.2)	-	38 (10.5)	-	0.99 (0.63 - 1.56)
Problem behaviours‡	53 (14.2)	-	48 (13.3)	-	1.06 (0.72 - 1.57)
Use of antipsychotic medication or physical restraints§	72 (19.4)	-	86 (23.8)	-	0.81 (0.59 - 1.10)
Indicators of depression or anxiety almost daily‖	56 (15.1)	-	50 (13.9)	-	1.05 (0.71 - 1.55)
Use of anti-anxiety medication¶	25 (6.7)	-	24 (6.6)	-	0.99 (0.57 - 1.74)
Use of bedtime sleeping pill¶	32 (8.6)	-	31 (8.6)	-	0.99 (0.61 - 1.63)
Other					
Partial or Full thickness skin ulceration (stage 2-4)	34 (9.1)	-	36 (10.0)	-	0.90 (0.56 - 1.45)
Urinary incontinence**	325 (87.4)	-	310 (85.9)	-	1.02 (0.87 - 1.19)

*Except for non-vertebral fracture, all safety outcomes are as recorded by nurses on the RAI-MDS 2.0 assessment closest to day 135 post-randomization.

† 'Deteriorated cognition relative to status 90 days earlier' (qualitative global assessment by nurse providing direct care).

‡ 'Behavioural symptoms that are present a minimum of 4 days per week and not easily altered in the last 7 days' (includes wandering, verbal abuse, physical abuse, socially inappropriate or disruptive behaviour and resisting care).

§In the last 7 days. Includes chair, trunk or limb restraints, but does not include bedrails.

‖In the last 30 days. Applies to 16 discrete mood and anxiety related observations of the resident, any one of which being listed as present 6 or more days per week would qualify.

¶On ≥3 of the last 7 days.

** ≥2 times per week.

© 2025 Garrison SR et al. *JAMA Network Open*.

eTable 8: Analysis of Unplanned All-cause Hospitalization/ED Visits as a Continuous Variable (Poisson Regression*), Counting All Events During Follow-up Period

**Outcome**	**Treatment (Bedtime) N = 394**	**Control (Morning) N = 382**	**Overall (N = 776)**	**Unadjusted RR (95% CI)**	**Adjusted RR (95% CI)**
**All-cause unplanned hospitalization/ED visit†**			**n (%)**		
Mean (SD)	0.48 (1.08)	0.49 (0.85)	0.49 (0.97)	0.94 (0.77 - 1.15)	0.87 (0.71 - 1.07)
Count, n(%)					
0	287 (72.8)	254 (66.5)	541 (69.7)		
1	65 (16.5)	89 (23.3)	154 (19.8)		
2	25 (6.3)	24 (6.3)	49 (6.3)		
3+	17 (4.3)	15 (3.9)	32 (4.1)		
Rate / 100 patient-yr	34.8	36.9	35.8		

*Model includes offset term for duration of follow-up for each person (natural logarithm of years of follow-up).

†Hospitalization/ED visits are combined into episodes of care so as not to count the same episode multiple times. Encounters within 1 day are considered the same episode. e.g., ED -> Hosp (admitted from ED) = 1 visit; Hosp -> Hosp (transfer) = 1 visit

© 2025 Garrison SR et al. *JAMA Network Open*.

eTable 9: Unadjusted Hazard Ratios for Primary and Secondary Outcomes

**Outcome**	**Treatment (Bedtime) N = 394**		**Control (Morning) N = 382**		**Unadjusted HR/RR (95% CI)**	**Adjusted HR/RR (95% CI)**
	**n (%)**	**Rate / 100 patient-yr**	**n (%)**	**Rate / 100 patient-yr**		
**Primary**						
Major adverse cardiovascular event	160 (40.6)	29.4	160 (41.9)	31.5	0.93 (0.75 - 1.16)	0.88 (0.71 - 1.11)
**Secondary – Efficacy**						
Primary Outcome Components						
All-cause mortality	157 (39.8)	28.7	157 (41.1)	30.7	0.94 (0.75 - 1.17)	0.89 (0.71 - 1.11)
Hospitalization for stroke	3 (0.8)	0.5	7 (1.8)	1.4	0.40 (0.10 - 1.57)	-
Hospitalization for MI/ACS	2 (0.5)	0.4	2 (0.5)	0.4	0.93 (0.13 - 6.51)	-
Hospitalization for HF	8 (2.0)	1.5	6 (1.6)	1.2	1.26 (0.44 - 3.63)	-
All-cause unplanned hospitalization/ED visit	107 (27.2)	22.6	128 (33.5)	30.0	0.76 (0.59 - 0.98)	0.74 (0.57 - 0.96)
**Secondary – Safety***						
Falls/Fractures						
Non-vertebral fracture	9 (2.3)	1.7	10 (2.6)	2.0	0.84 (0.34 - 2.07)	-
Fall in the past 30 days	53 (15.4)	-	54 (15.9)	-	0.97 (0.66 - 1.41)	0.97 (0.67 - 1.42)
Cognitive/Behavioural						
Deteriorated cognition[†]	32 (9.3)	-	35 (10.3)	-	0.90 (0.56 - 1.46)	0.92 (0.57 - 1.48)
Problem behaviours[‡]	50 (14.5)	-	42 (12.4)	-	1.17 (0.78 - 1.77)	1.13 (0.75 - 1.71)
Use of antipsychotic medication or physical restraints[§]	64 (18.6)	-	84 (24.7)	-	0.75 (0.54 - 1.04)	0.74 (0.53 - 1.03)
Indicators of depression or anxiety almost daily[‖]	56 (16.2)	-	48 (14.1)	-	1.15 (0.78 - 1.69)	1.11 (0.75 - 1.64)
Use of anti-anxiety medication[¶]	25 (7.2)	-	23 (6.8)	-	1.07 (0.61 - 1.89)	1.06 (0.60 - 1.87)

© 2025 Garrison SR et al. *JAMA Network Open*.

Use of bedtime sleeping pill¶	30 (8.7)	-	27 (7.9)	-	1.10 (0.65 - 1.84)	1.11 (0.66 - 1.87)
Other						
Partial or Full thickness skin ulceration (stage 2-4)	31 (9.0)	-	37 (10.9)	-	0.83 (0.51 - 1.33)	0.83 (0.51 - 1.33)
Urinary incontinence**	302 (87.5)	-	287 (84.4)	-	1.04 (0.88 - 1.22)	1.04 (0.88 - 1.22)

*Except for non-vertebral fracture, all safety outcomes are as recorded by nurses on the RAI-MDS 2.0 assessment closest to day 135 post-randomization.
† 'Deteriorated cognition relative to status 90 days earlier' (qualitative global assessment by nurse providing direct care).
‡ 'Behavioural symptoms that are present a minimum of 4 days per week and not easily altered in the last 7 days' (includes wandering, verbal abuse, physical abuse, socially inappropriate or disruptive behaviour and resisting care).
§In the last 7 days. Includes chair, trunk or limb restraints, but does not include bedrails.
||In the last 30 days. Applies to 16 discrete mood and anxiety related observations of the resident, any one of which being listed as
present 6 or more days per week would qualify.
¶On ≥3 of the last 7 days.
** ≥2 times per week.

© 2025 Garrison SR et al. *JAMA Network Open*.

eFigure 3: Effect of medication timing on all-cause death

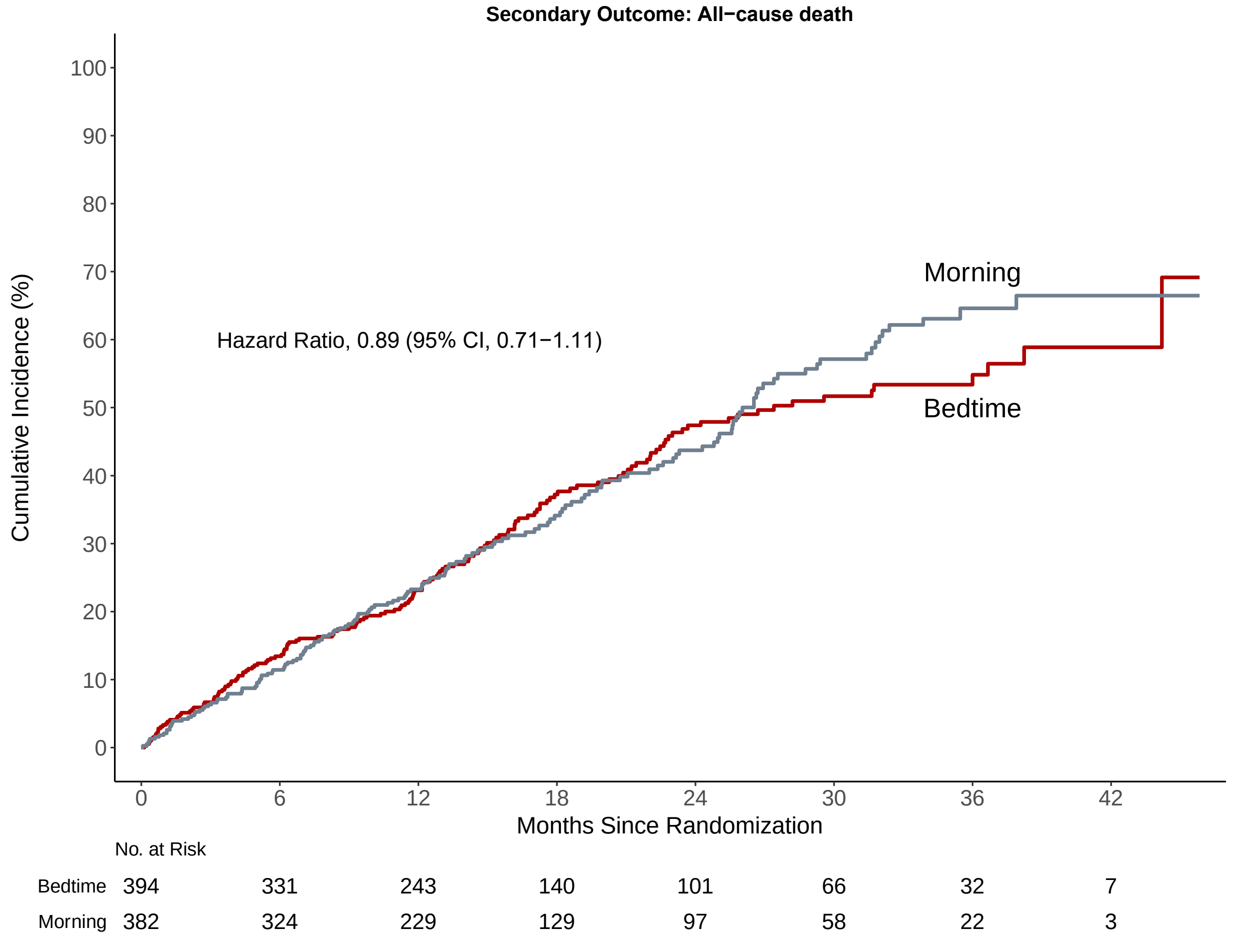

Figure shows the cumulative incidence of all-cause death for the comparison of bedtime versus usual care (largely morning) antihypertensive use. Baseline characteristics used as covariates to produce the adjusted hazard ratio shown were predefined at the protocol stage and included: Age <70 years, sex, facility/level of care specific mortality rate (prior 3 years), new admission (<60 days), old admission (>3 years), days hospitalized in prior 6 months, Charlson score, facility, total number of blood pressure medications, heart failure, chronic obstructive pulmonary disease, chronic kidney disease, stroke, and diabetes.

© 2025 Garrison SR et al. *JAMA Network Open*.
