# Supplementary material for: Bedtime vs Morning Antihypertensive Medications in Frail Older Adults: The BedMed-Frail Randomized Clinical Trial
Source: JAMA Netw Open. 2025 May 12;8(5):e2513812. doi: 10.1001/jamanetworkopen.2025.13812 (PMC12070236; doi:10.1001/jamanetworkopen.2025.13812)
Supplement: Supplement 3. — Data Sharing Statement [file jamanetwopen-e2513812-s003.pdf]

Data Sharing Statement

Garrison. Bedtime vs Morning Antihypertensive Medications in Frail Older Adults. *JAMA Netw Open*. Published May 12, 2025. doi:10.1001/jamanetworkopen.2025.13812

Data
Additional Information: National Library of Medicine; ClinicalTrials.gov; Trial registration number NCT04054648
Data available: Yes
Data types: Deidentified participant data
How to access data: The BedMed-Frail investigators wish to make a deidentified participant level dataset freely available to other researchers, and an official request for this to occur will be forwarded shortly to Alberta Health Services. However, the ability to make such a dataset publicly available is complicated by the trial's opt-out consenting methods. Without written informed consent from participants that describes the sharing of participant level data, it may not be possible for that data to leave the secure environment of the Alberta Health Services data steward. Once this has been determined, the ability to share participant level data and (if available) the steps to obtaining it, will be posted on the Pragmatic Trials Collaborative website (www.PragmaticTrials.ca).
When available: beginning date: 09-30-2025

Supporting Documents
Document types: None

Additional Information
Who can access the data: Anyone requesting the data
Types of analyses: Any purpose
Mechanisms of data availability: With a signed data access agreement
Any additional restrictions: N/A
